# Network-induced multistability through lossy coupling and exotic solitary states

**DOI:** 10.1038/s41467-020-14417-7

**Published:** 2020-01-30

**Authors:** Frank Hellmann, Paul Schultz, Patrycja Jaros, Roman Levchenko, Tomasz Kapitaniak, Jürgen Kurths, Yuri Maistrenko

**Affiliations:** 1 Potsdam Institute for Climate Impact Research (PIK), Member of the Leibniz Association, P.O. Box 60 12 03, D-14412 Potsdam, Germany; 2 Division of Dynamics, Łódź University of Technology, Stefanowskiego 1/15, 90-924 Łódź, Poland; 3 Faculty of Radiophysics, Electronics and Computer Systems, Taras Shevchenko National University of Kyiv, Volodymyrska St. 60, 01030 Kyiv, Ukraine; 4 Department of Physics, Humboldt University of Berlin, Newtonstr. 15, 12489 Berlin, Germany; 5 Saratov State University, Saratov, Russia; 6 Institute of Mathematics and Centre for Medical and Biotechnical Research, National Academy of Sciences of Ukraine, Tereshchenkivska St. 3, 01030 Kyiv, Ukraine

**Keywords:** Physics, Statistical physics, thermodynamics and nonlinear dynamics, Complex networks, Nonlinear phenomena

## Abstract

The stability of synchronised networked systems is a multi-faceted challenge for many natural and technological fields, from cardiac and neuronal tissue pacemakers to power grids. For these, the ongoing transition to distributed renewable energy sources leads to a proliferation of dynamical actors. The desynchronisation of a few or even one of those would likely result in a substantial blackout. Thus the dynamical stability of the synchronous state has become a leading topic in power grid research. Here we uncover that, when taking into account physical losses in the network, the back-reaction of the network induces new exotic solitary states in the individual actors and the stability characteristics of the synchronous state are dramatically altered. These effects will have to be explicitly taken into account in the design of future power grids. We expect the results presented here to transfer to other systems of coupled heterogeneous Newtonian oscillators.

## Abstract (web summary)

The design of future power grids with decentral control calls for a better understanding of the stability of synchronized networked systems. Here, Hellmann et al. show that the energy losses in coupled oscillators can significantly alter power grid dynamics by introducing solitary states in the network.

## Introduction

The power grid is a vast network connecting generators and consumers of electrical energy. Due to the ongoing energy transition, dynamical actors are becoming more numerous and heterogeneous and new dynamical phenomena are expected to occur in future power grids dominated by prosumers. This has brought the dynamical stability of the necessary 50/60 Hz synchronous state into sharp focus and spurred a large number of theoretical works on this topic recently^1–8^. It is known that these oscillator networks can be multistable and even support multiple synchronous states^9,10^. Strong perturbations can move the power grid dynamics out of the basin of attraction of the synchronous state^11–14^: synchrony collapses and a blackout is the likely result.

The question of the stability of synchronisation is not specific to power grids but is central to a wide range of systems, like coupled Josephson junctions and laser systems^15,16^, animal and bacterial flocking behaviour^17,18^, Huygens’s pendulum clocks^19^, crowd synchrony on London’s Millennium Bridge^20,21^ and chemical^22,23^ or mechanical oscillators^24,25^ as well as the networks of neurons^26^.

In this study, we show that the non-linear dynamics of oscillators in power grids is fundamentally altered by the resistance of the lines (i.e. transfer conductances) and the resulting energy losses. The line resistances translate to a phase-lagged coupling as in the Kuramoto–Sakaguchi (KS) model^27^. Such a coupling is characteristic for most systems that can be described as coupled phase oscillators, e.g. by using a phase reduction approach^28^. Various versions of the inertia-free KS model exhibit non-trivial multistability and complex synchronisation patterns^29–32^.

The presence of losses breaks the coupling symmetry and hampers a rigorous mathematical analysis, e.g. in terms of Lyapunov functions^33,34^. Thus, as a trade-off in favour of analysability, models are typically studied assuming a lossless regime and much less is known about the lossy case (e.g. refs. ^1,2,35,36^).

## Results

### Network-induced multistability

**Fig. 1:**
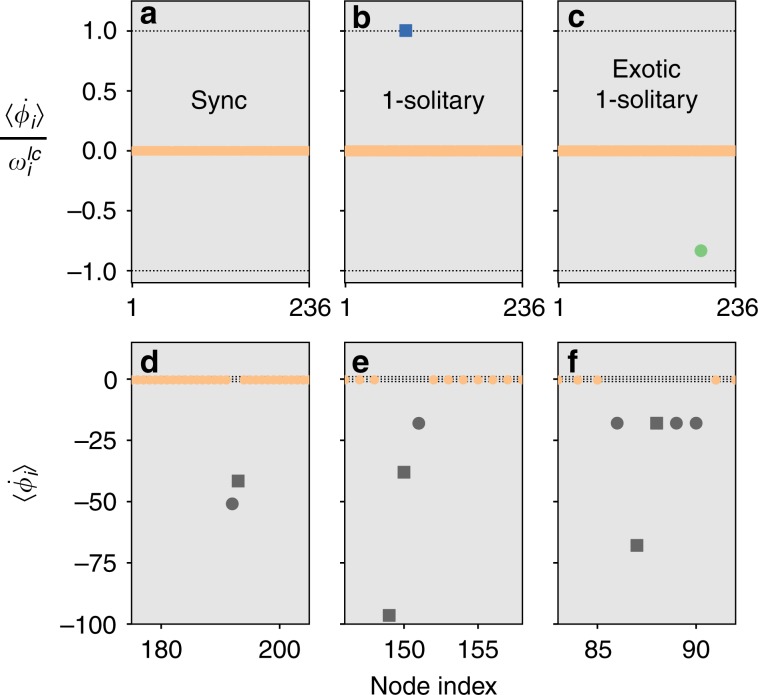
Classification of asymptotic states. The six panels depict the time-average $\langle {\dot{\phi }}_{i}\rangle$ of the instantaneous angular velocity vs. the node index *i*. The values in the top row are normalised to the respective natural frequency ${\omega }_{i}^{LC}=\frac{{P}_{i}}{{D}_{i}}$. **a** phase locking, **b** 1-solitary, **c** exotic 1-solitary, **d** 2-solitary, **e** 3-solitary and **f** a cluster that is desynchronised from the network but synchronised internally, with a solitary oscillator in it. The plots in **e**, **f** are restricted to a relevant subset of nodes for better visualisation. The asymptotic regimes correspond to different initial conditions of the Scandinavian grid with a 1:1 prosumer scenario, strongly coupled (K+) with standard damping (D) and *α* = 0.24.

In the following, we address the problem how a lagged coupling alters the multistability of the system with a focus on possible pathways of desynchronisation, particularly by a localised effective decoupling of one or several oscillators from the grid. The prototypical state for this scenario is the so-called 1-solitary state in which a single oscillator falls out of synchrony. In the lossless case, it decouples from the network and starts to rotate at its natural frequency. Figure 1 illustrates a variety of possible asymptotic states in oscillator networks characterised by their average phase velocity profile. That is, we sketch the time averaged phase velocity of the oscillators $\langle {\dot{\phi }}_{i}\rangle$, relative to their natural limit cycle frequency ${\omega }_{i}^{lc}$ for various scenarios. A solitary is given in Fig. 1b and phase locking in Fig. 1a.

We additionally find that the presence of losses can vastly increase the likelihood that a random perturbation hits the basin of attraction of a solitary state in many parameter regimes. Further, if a solitary state is created, it tends to destabilise the neighbouring nodes and cause their desynchronisation too.

It is important to emphasise that some parameter regimes and topologies in which synchronisation is completely stable without losses become multistable in the presence of even minimum losses. Remarkably we uncover novel exotic solitary states, illustrated in Fig. 1c, in which the oscillator that falls out of synchrony rotates against its natural frequency. This is due to the losses in the network leaving an imprint on the dynamics of the effectively decoupled oscillator. These states appear in all parameter regimes and topologies studied. The phenomenon seems to be completely robust and stable.

Besides the solitary states, a plethora of other asymptotic states exist in the system. Figure 1d–f illustrate the most prominent ones, namely multiple solitaries and other partial synchronised regimes.

These results show that losses are more important than expected when it comes to qualitative features of the non-linear behaviour of the system. Further, the properties of the network coupling can fundamentally alter the dynamics of the oscillators in the system, even in asymptotic states in which they effectively decouple. We, therefore, conclude that developing a detailed understanding of the non-linear network physics of lossy power grids is of great practical importance going forward.

### The model

To start with, we will now describe the system’s equation. For a more detailed discussion of the parameter regimes studied here, we refer the reader to the “Methods” section. In keeping with established practice in the theoretical physics and control engineering community, we will study the power grids frequency behaviour using the Kuramoto model with inertia, also known as the multi-machine swing equation. The phase *ϕ*_*i*_ of the complex voltage at the nodes is the main dynamical variable. From engineering practice, it is known that this equation accurately captures the short time behaviour of the synchronous machines used today^37,38^ but it also serves as a fairly general starting point for the study of future control dynamics^5^. It accounts for a proportional response of the frequency to power imbalance and the presence of inertia but neglects higher order internal dynamics at the nodes as well as variations in the voltage magnitude. In the reference frame co-rotating with the synchronous frequency, the equations for *n* nodes are given as:

1 $$	{H}_{i}{\ddot{\phi }}_{i}= \, {P}_{i}-{D}_{i}{\dot{\phi }}_{i}-\sum _{j=1}^{n}{P}_{ij}\ ,\\ 	{P}_{ij}= \, {K}_{ij}(\sin ({\alpha }_{ij})+\sin ({\phi }_{i}-{\phi }_{j}-{\alpha }_{ij}))\ .$$

Here *P*_*i*_ is the net power injected/consumed at node *i*, the damping coefficient *D*_*i*_ characterises the power’s response to frequency changes and will mostly be determined by the droop (i.e. proportional) control present at the nodes, *H*_*i*_ is the inertia constant and *P*_*ij*_ is the power injected at node *i* into the line connecting nodes *i* and *j*. This power is given in terms of the phase difference and the complex admittance ${Y}_{ij}=-i{K}_{ij}\exp (i{\alpha }_{ij})$, with *K* and *α* typically positive (see “Method” section). This is the inverse of the complex impedance, consisting of the reactance *X* and the resistance *R*: *Y*_*ij*_ = 1 ∕ (*R*_*ij*_ + *iX*_*ij*_). All quantities are in the per unit system, such that the voltage magnitude equals one. The lossless assumption is that the transmission lines are purely inductive, that is, *R*_*ij*_ = 0 and thus ${\alpha }_{ij}=\arctan ({R}_{ij}/{X}_{ij})=0$. We will be interested in understanding how the dynamical non-linear properties change as a function of *α*.

Note that we here chose to keep the system injection balanced with ∑_*k*_*P*_*k*_ = 0. This leads to a slight decrease of the synchronous frequency with increasing losses (see Supplemental Fig. 2). Alternatively, one can compensate for the losses in the injected power. This would make *P*_*k*_ dependent on *α* but would keep the coherent frequency constant. The latter approach is typically used in the power systems community, see e.g. ref. ^39^.

We consider different regimes by separately increasing the relative strength of the damping coefficient (denoted *D*, *D*+ and *D*++) and the coupling (denoted *K* and *K*+) and by considering the ratio of producers to consumers for a balanced prosumer system and today’s system (1:1 and 1:3, respectively). The producers and consumers are distributed randomly but in such a way as to be appropriate to the network structure. The detailed discussion of the parametrisation can be found in the “Methods” section.

It is understood that the swing equation does not provide a fully realistic picture of the behaviour of a power grid. Besides the assumptions noted above, the non-linear behaviour of generators requires more detailed internal models to capture additional effects. For detailed quantitative studies, more realistic models which consider line losses are required. However, it is generally assumed that the swing equation does faithfully represent the qualitative short term frequency dynamics and the stability of the synchronous state as for these purposes losses can be neglected. It is in this context that the lossless case has received considerable theoretical attention.

In contrast, we find that the qualitative dynamical effects due to losses can not be neglected for this type of study. In particular, they are considerably more important than a variety of other modelling assumptions that have been discussed in the literature. The effects are already very pronounced for currently most studied high voltage power grids (*α*_*ij*_ ≈ 0.24, see “Methods” section) and become a dominant factor for future decentralised energy production with much higher losses on medium voltage power lines and a high share of prosumers (*α*_*ij*_ ≈ 1.4).

### The Scandinavian grid

We begin by studying the finite-size perturbations at a randomly selected but fixed producing node in the well-studied Scandinavian power grid (see Supplementary Fig. 1 and the “Method” section) with *n* = 236 nodes and a mean degree of $\bar{d}=2.7$^12,40^.

**Fig. 2:**
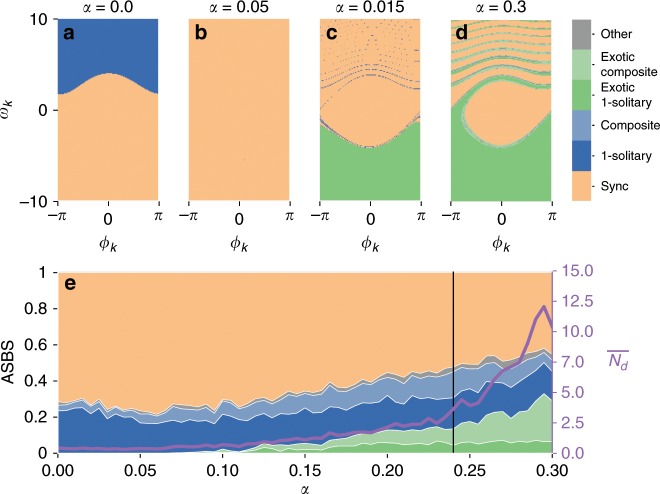
Phase space slices and ASBS for the Scandinavian power grid. In **a** we depict a slice of the phase space corresponding to phase *ϕ*_*k*_ and phase velocity *ω*_*k*_ of a randomly chosen producer node of the Scandinavian power grid in the prosumer scenario. The colour indicates the asymptotic state reached from running the system with standard parametrization from this point in phase space (all other nodes being in the synchronous state). They indicate synchrony (peach), a 1-solitary, (blue), an exotic 1-solitary (green), complex composite states containing multiple independent solitaries (i) excluding (light blue) or (ii) including exotic soliatries (light green) and others without solitary oscillators (grey). In **b** we plot the average size of the slice of the basin across all nodes (ASBS) for these classes of states. The right axis shows the expected number of desynchronised nodes $\overline{{N}_{d}}$ as a consequence of a random perturbation.

Figure 2a–d show the slices of phase space corresponding to a producing node for varying losses *α*. The colour indicates the asymptotic state reached after running the system with the chosen node in the plotted initial condition and the other nodes in the synchronous state.

We distinguish the system returning to the synchronous state (peach), a 1-solitary, with exactly one oscillator rotating out of synchrony in the direction of its natural frequency (blue), an exotic 1-solitary with the desynchronized oscillator rotating against its natural frequency (green) and complex composite states where at least one synchronous component co-exists with either multiple independent solitary oscillators excluding exotic solitaries (light blue) or multiple exotic solitaries/a mix of exotic and non-exotic solitaries (light green). There are other complex partially synchronised, cluster-synchronised and desynchronised states without solitaries, which we do not distinguish further (grey). The classes are mutually disjoint.

We immediately see a dramatic change in structure for the range of *α* studied. For a typical transmission grid as the Scandinavian one, the engineering textbooks give values of *α* ≈ 0.24 as realistic (see “Methods” section). Even much smaller values lead to a dramatic change in the basin structure. Increasing *α* = 0–0.05, the basin of solitary state disappears, only to reappear at *α* = 0.15 but mirrored as an exotic solitary, rotating contrary to the natural limit cycle. For higher values of *α*, other states start playing a role.

The proportion of the sync basin in the phase space slices in Fig. 2a–d has a direct interpretation in terms of the stability with respect to finite perturbations and has been referred to as single node basin stability^12^, which measures the probability for the whole system to converge to a particular asymptotic state after a finite random perturbation at a specified node. To quantify the multistability around the synchronous regime, we consider in Fig. 2e the average of these single node basin stabilities (ASBS) across all nodes of the network. The ASBS of the synchronous state is a proxy for the stability of the system with respect to realistic large perturbations that will typically be geographically localised in the power grid. The numerical procedure is described in the “Method” section.

We see that the ASBS of the synchronous state falls from roughly 0.75–0.5. This loss of stability is brought on by an increasing likelihood of reaching solitary states. Specifically, the presence of exotic solitary states seems to account for most of the decrease. After their onset at around *α* = 0.1, the basin of attraction of states containing solitaries rises to more than 25% at *α* = 0.3. We also show the average number of oscillators displaced from synchrony $\overline{{N}_{d}}$ following a random peturbation at a random node. This indicates that the perturbations desynchronise larger parts of the network as *α* increases.

In general, we consider ASBS a more meaningful measure compared to the approaches based on global perturbations since for power grids the realistic faults will typically be geographically localised in the network. For more detailed discussion of the probabilistic stability measures used here see the Methods section and Supplementary Note 2.

### The circular grid

**Fig. 3:**
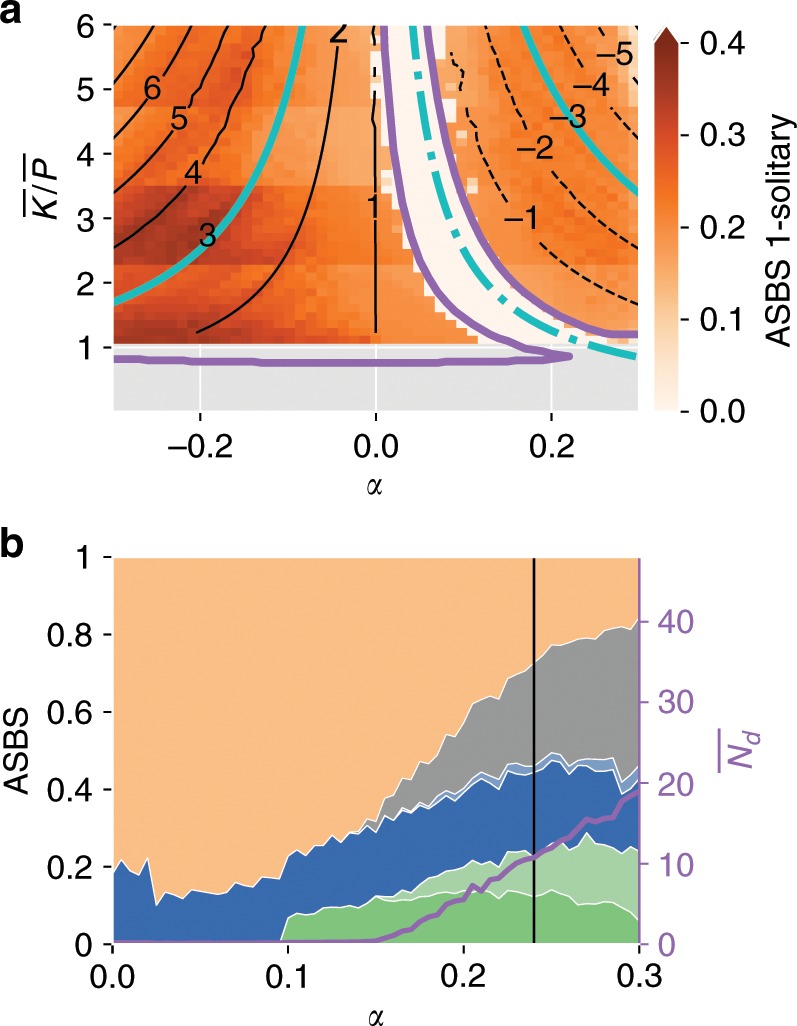
Parameter study and ASBS for the circle power grid. In **a** we show the *K*∕*P*-*α* parameter plane for solitary oscillations in the circle topology at a producer. The colour scale indicates the average single node basin stability ASBS of normal respectively exotic solitaries after a perturbation at a producer. The black lines give the mean phase velocity of the observed solitaries as an integer multiple of the natural limit cycle *ω*^lc^, positive for solid, negative for dashed line style. The cyan solid lines show the analytic location of the ${\langle \dot{\phi }\rangle }_{T}=3{\omega }^{\text{lc}}$ limit cycle from the decoupling model. The dash-dotted cyan line marks the respective hypothetical location of the ${\langle \dot{\phi }\rangle }_{T}=0$ limit cycle. Additionally, the solid purple lines mark the extend of the solitary existence regions determined by numerical state continuation (see Methods). **b** Shows the ASBS of the circle with standard parametrisation. The asymptotic states are coloured according to the legend in Fig. 2.

To understand the origin of (exotic) 1-solitary states better, we consider a symmetric circle topology (illustrated in Supplementary Fig. 1, see also ref. ^41^, where a much more homogeneous version of the system without power flows has been studied) with *n* = 50 nodes and next to nearest-neighbour coupling leading to a mean degree of 4. Prosumers with net power production and consumption are placed alternately on the circle. This system is not designed to reflect realistic networks directly but to gain maximum insight into the dynamical origins of solitary states. Note in particular that the network has no dead ends. We confirm that this symmetric setup reproduces the emergence of solitary basins. As Fig. 3b shows, the ASBS of the synchronous state decreases while that of solitary states rises, with exotic 1-solitaries emerging quite suddenly at *α* = 0.1. The overall ASBS of the synchronous state is lower in this system.

In Fig. 3a we show a systematic study of the ASBS 1-solitaries (not differentiating exotic and standard), and the relative frequency of the typical solitary oscillations that occur after a perturbation at a net producer, in the $\bar{K}/\bar{P}$-*α* parameter plane. Note that the data points below line $\bar{K}/\bar{P}\approx 1$ are excluded, since ASBS is not defined in the absence of the synchronous fixed point (see “Method” section). We also show nonphysical values of negative *α*. Due to the high symmetry of the setup these can be mapped by reflection (*ϕ* → −*ϕ*, *α* → −*α*) to the behaviour of net-consumer nodes. We see that there is a curved region in the middle where the ASBS goes to zero. We also see that the frequency at which the decoupled oscillator rotates changes considerably.

### Solitary state frequencies

We can gain a better understanding of the shifting frequency of these (exotic) 1-solitary states by considering the single machine infinite bus model. In the engineering literature (e.g. ref. ^42^), the standard analysis of the return to synchrony after a frequency event at a node neglects the back-reaction of the dynamics at node *i* on the other nodes, leading to the equation:

2 $$H\ddot{\phi }=P-D\dot{\phi }-{K}^{{\prime} }\sin (\alpha )-{K}^{{\prime\prime} }\sin (\phi -\alpha )\ ,$$

where *ϕ* denotes the phase difference between the solitary and the synchronous component. The total capacity of connecting lines ${K}^{{\prime} }$ and the effective coupling constant *K*^″^ can be derived directly from Eqn. (1) (see “Method” section).

When decoupling the single oscillator from the infinite bus (${K}^{{\prime} }={K}^{{\prime\prime} }=0$), the oscillator rotates freely with the frequency ${\omega }^{\text{lc}}:= \frac{P}{D}$. When the coupling is switched on, the system becomes bi-stable but this limit cycle persists and in the absence of losses its average frequency stays close to *ω*^lc^^12^. This can be seen as a simple model for solitary states^41,43^, where the infinite bus represents the remaining synchronous component.

We want to explore the assumption that the persistence of the limit cycle arises out of an effective decoupling due to the $\sin (\phi (t)-\alpha )$ term being averaged out. Let us assume that the frequency varies slowly, i.e. $\ddot{\phi }\approx 0$, and that $\dot{\phi }$ varies little around a constant value *ω*$^{*}$. Then we can derive a consistency condition for *ω*$^{*}$ by considering the time average of $\dot{\phi }$ over one period *T* = 2*π*∕*ω*$^{*}$:

3 $$	0\approx \, P-D{\omega }^{* }-K^{\prime} {\mathrm{sin}}(\alpha )-K^{\prime\prime} {\langle \sin ({\omega }^{* }t-\alpha )\rangle }_{T}\\ 	{\omega }^{* }\approx \, \frac{P}{D}-\frac{K^{\prime} }{D}{\mathrm{sin}}(\alpha ).$$

Thus we expect the limit cycle to be shifted to ${\langle \dot{\phi }\rangle }_{T}={\omega }^{* }={\omega }^{\text{lc}}\left(1-\frac{{K}^{{\prime} }}{P}\sin (\alpha )\right)$ in the presence of losses. Especially for a weakly loaded system with ${K}^{{\prime} }\gg P$ this shift can be huge and easily flips the sign of ${\langle \dot{\phi }\rangle }_{T}$.

The location of the shifted limit cycle as predicted by Eqn. (3) agrees well with the numerical evidence; the lines 3*ω*^lc^,  −3*ω*^lc^ and 0 for ${\langle \dot{\phi }\rangle }_{T}$ are marked in the systematic parameter plane study of Fig. 3a. We see that the decoupling-by-averaging picture matches perfectly the numerical results from the full system. In the presence of the losses the averaging of the energy exchange with the rest of the network in a highly oscillatory setting leaves an imprint.

We also see that the disappearance of the solitary and the later appearance of the exotic solitary state in the phase space slices Fig. 2a–d can be understood as ${\langle \dot{\phi }\rangle }_{T}$ being shifted close to zero and then beyond. This also suggests that both transitions from synchronisation to solitary oscillations are homoclinic bifurcations, which occur for the infinite bus model. This is supported numerically by a logarithmic scaling of the solitary’s oscillation period and further by the mean-field approximation (see Supplementary Note 1 and the discussion in ref. ^41^).

Finally, this suggests why increasing *α* will always lead eventually to a decrease in the size of the sync basin. If the shifted limit cycle is close to 0, it becomes entrained to the synchronous state and a solitary state is not possible. Increasing *α* can initially shift the limit cycles of net producers closer to 0, stabilising the system. This is observed in Fig. 3b. As ${K}^{{\prime} }> P$ though, the sign of the shifted limit cycle will eventually flip and the exotic solitary will exit the entrainment region and become stable. In the symmetric case of the circle this happens at the same *α* at all nodes. In contrast, in the Scandinavian grid these effects will be smeared out by the heterogeneous distribution of both ${K}^{{\prime} }$ and *P* in the system.

### Generality of the results

**Fig. 4:**
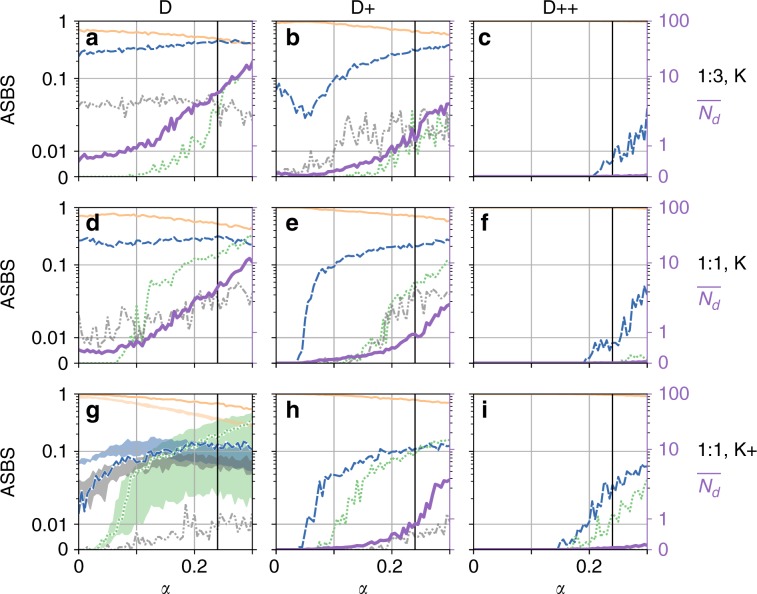
Average single node basin stability for different regimes. The panels depict the size of the single node basin for different asymptotic states in the Scandinavian grid: synchronisation (full line, peach), states containing exotic solitaries (dotted line, green), states containing normal solitaries but no exotic ones (dashed line, blue) and the remainder (dot-dashed line, grey), as a function of *α*. The columns refer to **a**–**g** standard (D) **b**–**h** strongly (D+) and **c**–**i** very strongly damped (D++) regimes. The first row **a**–**c** refers to the status-quo scenario 1:3 with standard coupling (K). The last two rows refer to the prosumer scenario 1:1 with **d**–**f** standard (K) and **g**–**i** strongly coupled regimes. The right axis in each subfigure shows the expected number of desynchronised nodes $\overline{{N}_{d}}$. For each value of *α*, the shares sum up to 1. Note the logarithmic scale of the ordinate. **g** Also includes runs from an ensemble of ten randomly generated power grids with a comparable structure^44^ (details in “Method” section). Pictured is the range between the 25% and 75% quantiles of the distributions for each *α*. The right axis in all subfigures shows the expected number of desynchronised nodes $\overline{{N}_{d}}$ in a solid purple line.

To understand the robustness of the described phenomenon, we study a variation of parameter regimes for the Scandinavian topology as well as one regime on different synthetic network topologies. The results are given in Fig. 4; further results are given in Supplementary Figs. 3 and 4. To reduce the complexity of our presentation Fig. 4, does not distinguish 1-solitaries from the composite states containing the solitaries. We only distinguish (possibly composite) states containing an exotic solitary in green and states containing solitaries but no exotic solitaries in blue.

We study three variations of the model. First we study the case of a lightly loaded system with large $\bar{K}/\bar{P}$. Then we consider the system with more aggressive droop control and a larger damping coefficient ${\bar{D}}^{2}/\bar{P}\bar{H}$. Finally, we also study a producer to consumer ratio of 1:3 that more closely resembles the situation in today’s high voltage transmission grids. We also consider other realistic topologies generated from the synthetic network ensemble^44^ (see details in “Method” section).

We find that in all studied scenarios, normal or exotic solitaries start appearing more frequently as we increase *α*. This is especially true in the cases where the system has been relatively stable for *α* = 0. The ASBS of the synchronous state decreases everywhere with increasing losses. We find that a very aggressive droop can delay significantly the onset of solitary states.

For all regimes, there is a strong qualitative difference in the asymptotic structure between the lossless case *α* = 0 and the realistic values for *α*. An extremely strong droop control, which leads to a very stable system as far as the frequency dynamics are concerned, can significantly reduce the ASBS of solitaries at realistic values of *α*. However, for a weakly loaded system, the solitaries appear even for this case with a proportion of up to 10%.

The numerical continuation of solitary states in the $\bar{K}/\bar{P}$- *α* parameter plane suggests that, while the existence region for exotic solitaries is bounded by a maximum coupling strength, this is not the case for normal solitaries (see Fig. 3a and Supplementary Fig. 7). Hence, while the bi-modal state, where all nodes are desynchronised and oscillate close to their natural frequency *ω*^lc^, exists only for moderately small couplings *K*, solitary states persist even for coupling strengths much beyond realistic values. Due to the shape of the existence region of solitary states, an increase in the coupling at fixed *α* > 0 can even lead to exotic solitaries coming into existence and hence further destabilises the system, contrary to intuition.

Finally, we consider an ensemble of 10 different synthetic topologies with the same size and similar properties than the Scandinavian topology. The synchronous regime is consistently less stable than that in the actual Scandinavian grid due to an abundance of complex composite partially synchronised states. We used the *K*+ parametrisation to compensate this fact. The result is shown in Fig. 4g, where the distribution of ASBS across the sample from the ensemble is characterised by its 25 and 75% quantiles. We see that a qualitatively similar behaviour occurs for all topologies, especially the onset of exotic solitaries is reproduced consistently.

## Discussion

For all studied parameter regimes—including the case of a realistically parametrised Scandinavian system—we observe a significant reduction of the size of the sync basin when going to realistic values of losses. To our knowledge, this very generic phenomenon has not been observed in literature before. It has far reaching implications for the design and control of future power systems. Our results show that it is not possible to use theoretical results on the stability of systems without losses (*α* = 0) in the design of future power systems as it is. The losses fundamentally alter the dynamical behaviour of the coupled systems.

A strong control regime, with an extremely high damping ratio ${\bar{D}}^{2}/\bar{P}\bar{H}$ of a hundred times that of the standard value (see “Methods” section), can stabilise the system up until *α* ≈ 0.2. Beyond this, however, for realistic values of *α* ≈ 0.24 the sync basin is slowly decreasing. Hence, for future distribution grids with *α* much larger than 0.24 even such a control effort is likely not sufficient to eliminate the multistability induced by the losses.

Further, as noted above, a mere increase of coupling, e.g. by improving transmission capacities in a power grid, is not sufficient to prohibit non-synchronous solitary oscillations either. In strongly damped situations it can even enhance their basin.

This leads also to a novel insight into the relative importance of damping and coupling. The classic results on the synchronisation of oscillators^1,2^ give conditions that depend primarily on *K* but not *D*. We typically speak of critical coupling strength. In contrast, here we see that, when taking the losses into account, *D* is considerably more important.

The study of lossy coupling also has implications to applications in engineering. While case studies typically work with the physically correct equations involving losses (e.g. ref. ^45^) it is also typical to neglect the losses of e.g. coupled two-area systems^46^. It remains unclear whether this is justified. Novel control strategies based on basin structure of the lossless case, like these in ref. ^47^, will certainly need to be revised. The degree to which the presence of losses also affects the transient and stochastic behaviour of the power grid is also a subject of ongoing research^48,49^.

The fact that 1-solitaries can be understood from the single machine infinite bus model means that further investigation—including the experiments and analytics—can begin on simplified systems and the effect can be cleanly isolated from the pronounced local topological effects present in real world topologies. In particular, solitary states persist even in the mean-field limit with an all-to-all coupling. Analytic calculations for these cases are stated in the Supplementary Discussion.

Our results on the conceptual importance of losses also confirm the experience with the static, linearized behaviour of the system. The fixed point solutions of Eqn. (1) correspond to solutions of the stationary power flow problem. For large networks, this is a challenging problem in itself and is often studied using linearizations known as the DC approximation. It has been found that building the approximation in such a way as to take losses into account, dramatically improves their accuracy^39,50–52^.

Finally, as noted before, the model studied here can be equivalently seen as representing generic Newtonian oscillators, coupled by the lowest Fourier mode. In fact, when approximating periodic or chaotic systems as phase oscillators (see e.g. ref. ^28^), the parameter *α* appears generically. We, therefore, expect the phenomenon described here to pertain to a wide range of non-linear heterogeneous oscillator systems.

## Methods

### Network topologies

Typical values for ohmic losses and reactances in power grids^53^ are given in Table 1.

**Table 1:** Typical line parameters.

Line	Transmission	Distribution	Low voltage
R [Ω km^−1^]	0.1	0.4	0.5
X [Ω km^−1^]	0.4	0.3	0.08
*α*	0.24	0.93	1.41

Thus the regime studied in this work covers transmission lines, where the lossless approximation has been taken to be valid.

Our main application is the Scandinavian power grid^12,13^. In Supplementary Fig. 1, we schematically depict the network topology of the Scandinavian grid and the circle with next-to-nearest-neighbour coupling. The latter will be discussed in the Supplementary Discussion in more details. The data set of the Scandinavian grid^12^ contains *n* = 236 substations (nodes) and *m* = 320 transmission lines (edges) in between, corresponding to an average degree of 2.7. This sparse network structure is characteristic for most power grid data sets^44^. The Scandinavian data set has been originally extracted from the map of the “Interconnected network of Northern Europe” published by ENTSO-E in 2014 and contains the approximate lengths of the transmission lines (110 kV and higher) due to the geographic information but not the specific line parameters. For the latest version see: https://docstore.entsoe.eu/Documents/Publications/maps/2019/Map_Northern-Europe-3.000.000.pdf. For our analysis, we consider the giant component of the network; parallel circuits are combined to single edges in the network, avoiding multiple edges. The data set has been studied previously w.r.t. the synchronous regime’s stability under single-node perturbations^12^ as well as transient survivability^13^.

### Synthetic network ensemble

In Fig. 4g we contrasted the results for the Scandinavian topology with 10 realisations of a synthetic network ensemble^44^ for spatially-embedded infrastructure networks. The single realisations are created in a random growth process that is oriented on the historical evolution of (ultra)-high voltage electrical transmission grids. The central idea is that during the growth there is a trade-off between building costs and additional redundancy for security aspects. The preference is controlled via *r* parameter. An emerging node connects to at least one neighbour or splits an existing line in a preferential attachment rule. Locations are drawn uniformly at random from the unit square but real data could be used as well. Here we have applied the model parameters (*N*, *N*_0_, *p*, *q*, *r*, *s*) = (236, 1∕5, 3∕10, 1∕3, 1∕10) which have previously been shown to create networks statistically similar to e.g. the Scandinavian topology^54^. This set of parameters is, however, not optimised in a particular way but rather an informed guess. This is a part of ongoing research. Still, the ensemble realisations lie in the range of observations from common data sets^44^ and provide us with a tool to test hypotheses over a network ensemble.

### Dynamical regimes

Consider the infinite busbar model Eqn. (2). The bifurcation structure of the system is invariant under time re-parametrisation and thus there are two characteristic quantities in the system. Meaningful choices for invariant parameter combinations are $\frac{K}{P}$, which characterises the line load and $\frac{{D}^{2}}{PH}$ which describes the strength of the droop control relative to the power in-feed.

To parametrise the networked system Eqn. (1), which generally has highly heterogeneous parameter sets, we introduce the network averages $\bar{P}:= \frac{1}{n}{\sum }_{i}| {P}_{i}|$, $\bar{K}:= \frac{1}{m}{\sum }_{i,j}{K}_{ij}$ and $\bar{D}:= \frac{1}{n}{\sum }_{i}{D}_{i}$. Without loss of generality, we fix *H*_*i*_ ≡ 1 for all nodes. Furthermore, we assume homogeneous damping ${D}_{i}\equiv \bar{D}$.

In analogy to the infinite busbar model, where these quantities reduce to the invariants, we can characterise the parametrisations by average line loading $\bar{K}/\bar{P}$ and damping/droop control strength ${\bar{D}}^{2}/\bar{P}\bar{H}$. We consider two regimes for the line loading given in Table 2.

**Table 2:** Coupling regimes considered.

	Standard	Strongly coupled
	(K)	(K+)
$\bar{K}/\bar{P}$	6	18

Similarly, we also study a strongly damped regime with droop control driving the system to the fixed point and an extremely strong droop control (i.e. overdamped regime). The values are given in Table 3.

**Table 3:** Damping regimes considered.

	Standard	Strongly damped	Very strongly damped
	(D)	(D+)	(D++)
${\bar{D}}^{2}/\bar{P}\bar{H}$	1/100	1/10	1

The values commonly studied in the basin stability literature^12,40,54^ are *H* = 1, ∣*P*∣ = 1, *D* = 0.1 and *K* = 6, with a producer/consumer ratio of 1:1. In our terminology, this corresponds to standard line loading and standard control in a prosumer dominated power grid.

In this paper, we select a net producer to net consumer ratio of 1:1 for the prosumer case and 1:3 as a typical value obtained e.g. for European high-voltage transmission systems^55^, see Table 4.

**Table 4:** Producer to consumer ratios considered.

	Prosumer	Status Quo
	(1:1)	(1:3)
$\frac{\#\,\text{Net Producers}}{\#\text{Net Consumers}\,}$	1/1	1/3

Taking *P*_*i*_ to be constant in each group, the constraints ∑_*i*_ *P*_*i*_ = 0 (power balance) and $\bar{P}=1$ fix the *P*_*i*_ in a way that is comparable to the standard parameters above. A random dispatch is generated as follows, *P*_*i*_ are allocated randomly to the nodes in the network ten times, then the dispatch with the best synchronisation condition^1,2^ is chosen. This is a trade off between random dispatch and the dispatch that is designed to be compatible with the grid infrastructure.

The underlying assumption of this procedure is that while renewable energies mix up where the energy is produced and the actual dispatch will be much more varied than today, they will be integrated into the power grid in a manner appropriate to the existing infrastructure. Therefore, their position will not be arbitrary but somewhat optimized to the network.

For the circle, the producers and the consumers are placed alternately.

Note that, with $\bar{P}$ given, the ratio $\bar{K}/\bar{P}$ fixes the average line admittance (The variation of voltage magnitudes is not considered in the model Eqn. (1).). The individual values are then determined by the link length distribution and the specific impedance. Analogously, ${K}_{ij}\equiv \bar{K}$ for the circle topology.

The parametrisation is typically used in the theoretical physics literature in order to study network and multistability effects expected to be relevant in future power grids. It is not expected to be a very close representation of current power grids but to reveal structural features of the type of dynamics expected in future power grids. As the observed phenomenon is highly generic and depends primarily on the physics of the coupling, we expect it though to occur in all parametrisations of the swing equation.

### Derivation of the power flow equation

Here, we derive the lossy power flow equation (Eqn. 2). *P*_*ij*_ should be the outgoing flow at the node such that the power flow equation reads *P*_*i*_ = ∑_*j*_*P*_*ij*_. According to Ohm’s law, the current on a line is given by $\frac{{V}_{i}-{V}_{j}}{{R}_{ij}+i{X}_{ij}}$, (if the own voltage is higher, the current, and hence the energy flows away) or *Y*_*ij*_(*V*_*i*_ − *V*_*j*_). As *R*_*ij*_ and *X*_*ij*_ are positive, *Y*_*ij*_ has a positive real part and a negative imaginary part. The Laplacian *L*_*ij*_  =  *δ*_*ij*_∑_*k*_*Y*_*ik*_ − *Y*_*ij*_ then provides us with the total outgoing current: *I*  = *L* ⋅ *V*. To connect with the literature written in terms of phase lags *α*, we set *Y* =  −*iKe*^*iα*^. Then *Ke*^*iα*^ = *iY* has a positive real part and a positive imaginary part. Hence *K* and *α* are positive.

Using ${V}_{i}={e}^{i{\phi }_{i}}$, ${V}_{i}{V}_{i}^{\star }=1$, the power flow can then be written as follows:

4 $$\sum_{j}{P}_{ij}	= \, \Re ({V}_{i}{I}_{i}^{\star })=\Re \left(\sum _{j}{V}_{i}{L}_{ij}^{\star }{V}_{j}^{\star }\right)\\ 	= \, \Re \left({V}_{i}{V}_{i}^{\star }\sum _{k}{(-i{e}^{i{\alpha }_{ik}}{K}_{ik})}^{\star }\right)-\Re \left(\sum_{j}{V}_{i}{(-i{e}^{i{\alpha }_{ij}}{K}_{ij})}^{\star }{V}_{j}^{\star }\right)\\ 	= \, \Re \left(i{V}_{i}{V}_{i}^{\star }\sum_{k}{e}^{-i{\alpha }_{ik}}{K}_{ik}\right)-\Re \left(i\sum_{j}{V}_{i}{e}^{-i{\alpha }_{ij}}{K}_{ij}{V}_{j}^{\star }\right)\\ 	= \, -\Im \left(\sum_{k}{K}_{ik}{e}^{-i{\alpha }_{ik}}\right)+\Im \left(\sum_{j}{K}_{ij}{e}^{i({\phi }_{i}-{\phi }_{j}-{\alpha }_{ij})}\right)\\ 	= \, -\sum_{k}{K}_{ik}\sin (-{\alpha }_{ik})+ \sum_{j}{K}_{ij}\sin ({\phi }_{i}-{\phi }_{j}-{\alpha }_{ij})\\ 	= \, \sum_{j}{K}_{ij}\left(\sin ({\alpha }_{ij})+\sin ({\phi }_{i}-{\phi }_{j}-{\alpha }_{ij})\right)$$

### Synchronous regime with varying losses

In Supplementary Fig. 2 we depict the shift of the synchronous operating point under an increase of losses which appear as the phase shift *α* in Eqn. (1). The simulation has been performed for the Scandinavian power grid in the (1:1, K, D+)-setup of Fig. 4e. As outlined in the “Methods” section, we consider parametrisations under the constraint ∑_*j*_*P*_*j*_  = 0, i.e. a balanced pattern of consumption and generation. The droop control then corrects for the losses on the lines. As we deal with parametrizations in the high-voltage system these tend to be small, as can be seen in Supplementary Fig. 2a. Then, we can calculate (see ref. ^56^) the coherent frequency *ω*_global_ of the synchronous regime as the function of the phase lag *α* in terms of the losses on the lines:

$$	0= \, \sum _{i}\left({P}_{i}+{D}_{i}\ {\omega }_{\mathrm{global}}+\sum _{j}{P}_{ij}\right)\\ 	{\omega }_{\mathrm{global}}\sum _{i}{D}_{i}= \, -\sum _{ij}{P}_{ij}\\ 	{\omega }_{\mathrm{global}}\sum _{i}{D}_{i}= \, -\sum _{ij:i < j}({P}_{ij}+{P}_{ji})\\ 	{\omega }_{\mathrm{global}}= \, -\frac{\sum _{ij:i< j}{P}_{ij}^{\mathrm{loss}}}{\sum _{i}{D}_{i}}$$

the losses on the line ${P}_{ij}^{\mathrm{loss}}$ can be calculated in terms of the phase difference $\Delta {\phi }_{ij}^{* }={\phi }_{i}^{* }-{\phi }_{j}^{* }$ as:

$${P}_{ij}^{\mathrm{loss}} 	= \, {P}_{ij}+{P}_{ji}\\ \,	= K\sin (\alpha )+K\sin (\Delta {\phi }_{ij}^{* }-\alpha ) +K\sin (\alpha )+K\sin (\Delta {\phi }_{ji}^{* }-\alpha )\\ 	= \, K\sin (\alpha )+K\sin (\Delta {\phi }_{ij}^{* }-\alpha )-K\sin (\Delta {\phi }_{ij}^{* }+\alpha )\\ 	=\, 2K\sin (\alpha )(1-\cos (\Delta {\phi }_{ij}^{* }))$$

they vanish with *α* = 0 and when there is no power flowing on the line $\Delta {\phi }_{ij}^{* }=0$.

### Infinite busbar reduction

Our aim is to rewrite Eqn. (1) for a single node *s*, replacing the power flow term by an effective coupling, i.e.

5 $${H}_{s}{\ddot{\phi }}_{s}+D{\dot{\phi }}_{s}={P}_{s}-{K}_{s}^{{\prime} }\sin \alpha -{K}_{s}^{{\prime\prime} }(\phi )\sin ({\phi }_{s}-{\hat{\phi }}_{s}(\phi )-\alpha )\ .$$

Here, ${K}_{s}^{{\prime} }:= {\sum }_{j}{K}_{sj}$ is just the weighted degree of node *s* corresponding to the total capacity of lines connecting *s* to the remaining network. The local mean-field parameters ${K}_{s}^{{\prime\prime} }$ and ${\hat{\phi }}_{s}$ are a function of the phases of all nodes in the neighbourhood of *s* and hence are time-dependent in general. They are defined via the relation

6 $${K}_{s}^{{\prime\prime} }\exp (i{\hat{\phi }}_{s}):= \sum_{j\ne s}{K}_{sj}\exp (i{\phi }_{j})\ .$$

We can calculate the magnitude ${K}_{s}^{{\prime\prime} }$ explicitly:

7 $${K}_{s}^{{\prime\prime} }=\sqrt{{\sum}_{j\ne s}{\sum }_{l\ne s}{K}_{sj}{K}_{sl}\cos ({\phi }_{j}-{\phi }_{l})}\ .$$

Now in the infinite busbar assumption we neglect the reaction of the remainder of the network on the modelled node *s*, thus we assume ${K}_{s}^{{\prime\prime} }$ and ${\hat{\phi }}_{s}$ to be constant and given by the values in the steady state: ${\forall }_{j\ne s}{\phi }_{j}={\phi }_{j}^{* }$, such that ${K}_{s}^{{\prime\prime} }={K}_{s}^{{\prime\prime} }({\phi }^{* })$ and ${\hat{\phi }}_{s}={\hat{\phi }}_{s}({\phi }^{* })$. The approximation is justified when the inertia of the overall system is much larger than that of *s* (hence the name infinite busbar approximation), i.e. for large (infinite) system size. Then we can define new coordinates $\phi := {\phi }_{s}-{\hat{\phi }}_{s}$ and arrive at an effective equation in the form of Eqn. (2), the infinite busbar model. Opposed to an uncoupled oscillator, the infinite busbar is possibly multistable. Especially in the parameter regimes analysed here, a fixed point corresponding to *ϕ*_*s*_ being locked to the synchronous cluster and a limit cycle corresponding to the solitary coexist^57,58^.

### Probabilistic stability analysis

The sampling-based evaluation of probabilistic properties of dynamical systems^11–13,54,59–61^ is the main numerical method we apply here. In particular, basin stability is the probability that a system converges to a specific attractor given a distribution of random finite perturbations. This can be regarded as a volume measure of the attraction basin. In the context of synchronisation it has been popularised as the ‘size of the sync basin’^62^. A direct evaluation of the basin geometry is computationally not feasible in high-dimensional systems. Even if the set is known to be convex, recent algorithms scale with ${\mathcal{O}}({d}^{4})$ where *d* is the phase space dimension^63^. Instead, a Monte Carlo sampling yields efficient estimates of the volume, which standard error does not depend on *d*^11^.

Compared with mathematical methods based on Lyapunov functions, the advantage and draw-back of sampling based methods is that they are not sensitive to worst case scenarios. Instead, they provide accurate results on the typical behaviour of a dynamical system, even where analytic results are hard to achieve or not available.

The perturbations are drawn uniformly at random from a finite box centred around the synchronous state. The synchronous state itself changes with *α* and thus the box of initial conditions does, too. Supplementary Fig. 2 illustrates the variation of a synchronous state.

We use network-local perturbations where perturbations are localised at a particular node. Our corresponding stability measure, the average single node basin stability ASBS, i.e. the probability to converge to a particular state after a finite random perturbation at a randomly drawn node. In particular, we consider 1000 perturbations for each value of *α*, drawn uniformly at random from $\Delta {\dot{\phi }}_{i}\in [-10\,{\mathrm{Hz}},+10\,{\mathrm{Hz}}]$ and $\Delta {\phi }_{i}\in [{\phi }_{i}^{* }-\pi ,{\phi }_{i}^{* }+\pi ]$. Here, the ${\phi }_{i}^{* }$ denote the phase values at the synchronous fixed point, i.e. ASBS is determined by perturbing a randomly chosen node’s degrees of freedom away from the fixed point while leaving the remaining nodes synchronised. This particular choice of perturbations is the key difference to the determination of global basin stability^11^. Supplementary Note 2 discusses this in more details.

### Continuation study

To perform a numerical continuation of solitary states in the parameter plane (see Fig. 3 and Supplementary Fig. 7), we integrated the system with a Cash–Karp method (error *e* = 10^−4^) for *T* = 5 × 10^3^ s under the influence of small noise (amplitude *d* = 10^−5^) to ensure transverse stability. *T* is chosen large enough to exclude transient solitary desynchronisation. From a starting point, one of the parameters is varied in steps of *Δα* = 0.01 respectively *ΔK* = 0.1 until the solitary oscillation ceased to exist. Then, the last step is reversed and the algorithm adds one step to the other parameter, tracking a bifurcation line.

## Supplementary information

Supplementary Information

## Data Availability

Simulations were performed using the Scipy package^64^ and the pyBAOBAP package available at https://gitlab.pik-potsdam.de/hellmann/pyBAOBAP. Scipy uses the LSODA solver of the odepack Library^65^. All code and data will be published open source at https://gitlab.pik-potsdam.de/pschultz/lossysolitaryor is available upon request. The algorithm used to create the synthetic network topologies in Fig. 4g is available at https://gitlab.com/luap-public/random-powergrid.
