# Supplementary material for: Network-induced multistability through lossy coupling and exotic solitary states
Source: Nat Commun. 2020 Jan 30;11:592. doi: 10.1038/s41467-020-14417-7 (PMC6992754; doi:10.1038/s41467-020-14417-7)
Supplement: Supplementary file 1 — Supplementary Information [file 41467_2020_14417_MOESM1_ESM.pdf]

Supplementary Information
Network-induced multistability through lossy coupling and exotic solitary states

Frank Hellmann,[1,*] Paul Schultz,[1] Patrycja Jaros,[2] Roman Levchenko,[3]
Tomasz Kapitaniak,[2] Jürgen Kurths,[1,4,5] and Yuri Maistrenko[2,1,6]

[1]*Potsdam Institute for Climate Impact Research (PIK), Member of the Leibniz Association, P.O. Box 60 12 03, D-14412 Potsdam, Germany*
[2]*Division of Dynamics, Łódź University of Technology,Stefanowskiego 1/15, 90-924 Łódź, Poland*
[3]*Faculty of Radiophysics, Electronics and Computer Systems, Taras Shevchenko National University of Kyiv, Volodymyrska St. 60, 01030 Kyiv, Ukraine*
[4]*Department of Physics, Humboldt University of Berlin, Newtonstr. 15, 12489 Berlin, Germany*
[5]*Saratov State University, Saratov, Russia*
[6]*Institute of Mathematics and Centre for Medical and Biotechnical Research, National Academy of Sciences of Ukraine, Tereshchenkivska St. 3, 01030 Kyiv, Ukraine*

I. SUPPLEMENTARY FIGURES

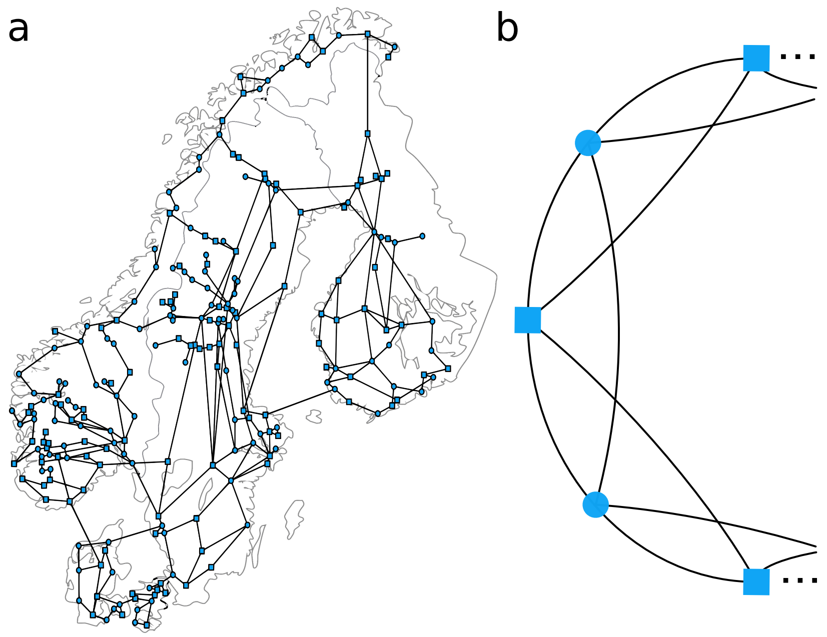

Supplementary Figure 1: **Network models. a** The Scandinavian (extra-)high voltage transmission grid. **b** A circle topology with coupling to next-nearest neighbours, i.e. a coupling radius of $R = 2$. In both cases, squares denote net consumers ($P < 0$) and circles net producers ($P > 0$).

In the Methods section, we have discussed the numerical procedure of the average single-node basin stability estimation and motivated the selection of different parameter regimes for Eqn. 1. For the Scandinavian and the circle topology we have performed additional simulations for all regimes and report them here in an overview.

Supplementary Figure 3 accompanies the results of Fig. 4 in the main text with additional simulations of (1:3, K+)-parametrisations. Note that here we plot the ASBS results on a linear scale that is cut-off at 0.6 to obtain an alternative visualisation also of the regimes with low ASBS. Furthermore, Supplementary Figure 3j contains the results of Fig. 2e.

---

* hellmann@pik-potsdam.de

(a) Instantaneous frequency (b) Phases

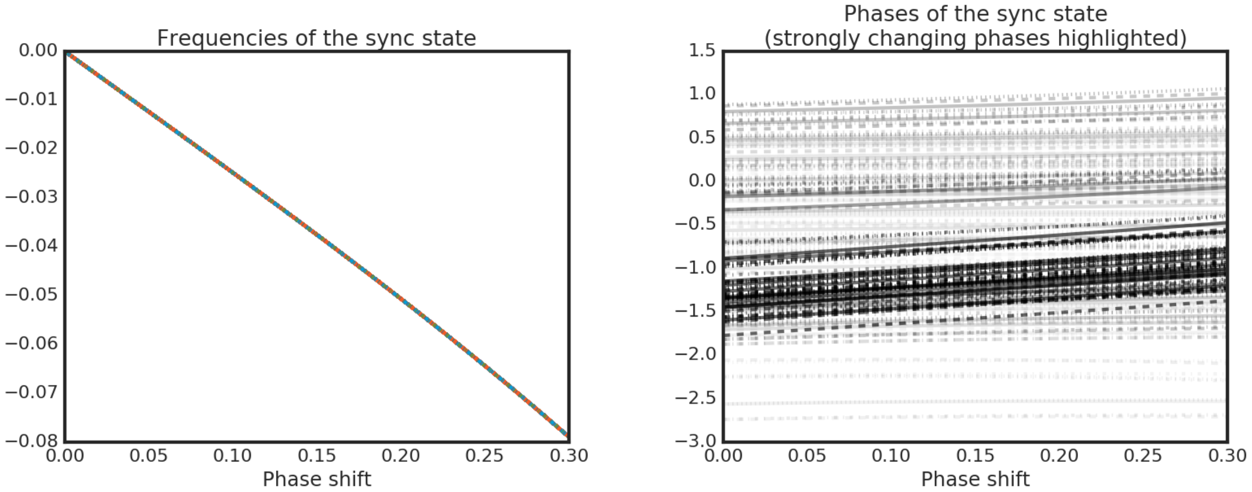

Supplementary Figure 2: **Synchronous regime.** Variation of the synchronous frequency ω_i^* (**a**) and phases ϕ_i^* (**b**) for all nodes i with increasing phase shift α.

Analogous to the previous figure, Supplementary Figure 4 reports the ASBS results for the circle topology. Supplementary Figure 4g contains the results of Fig. 3b.

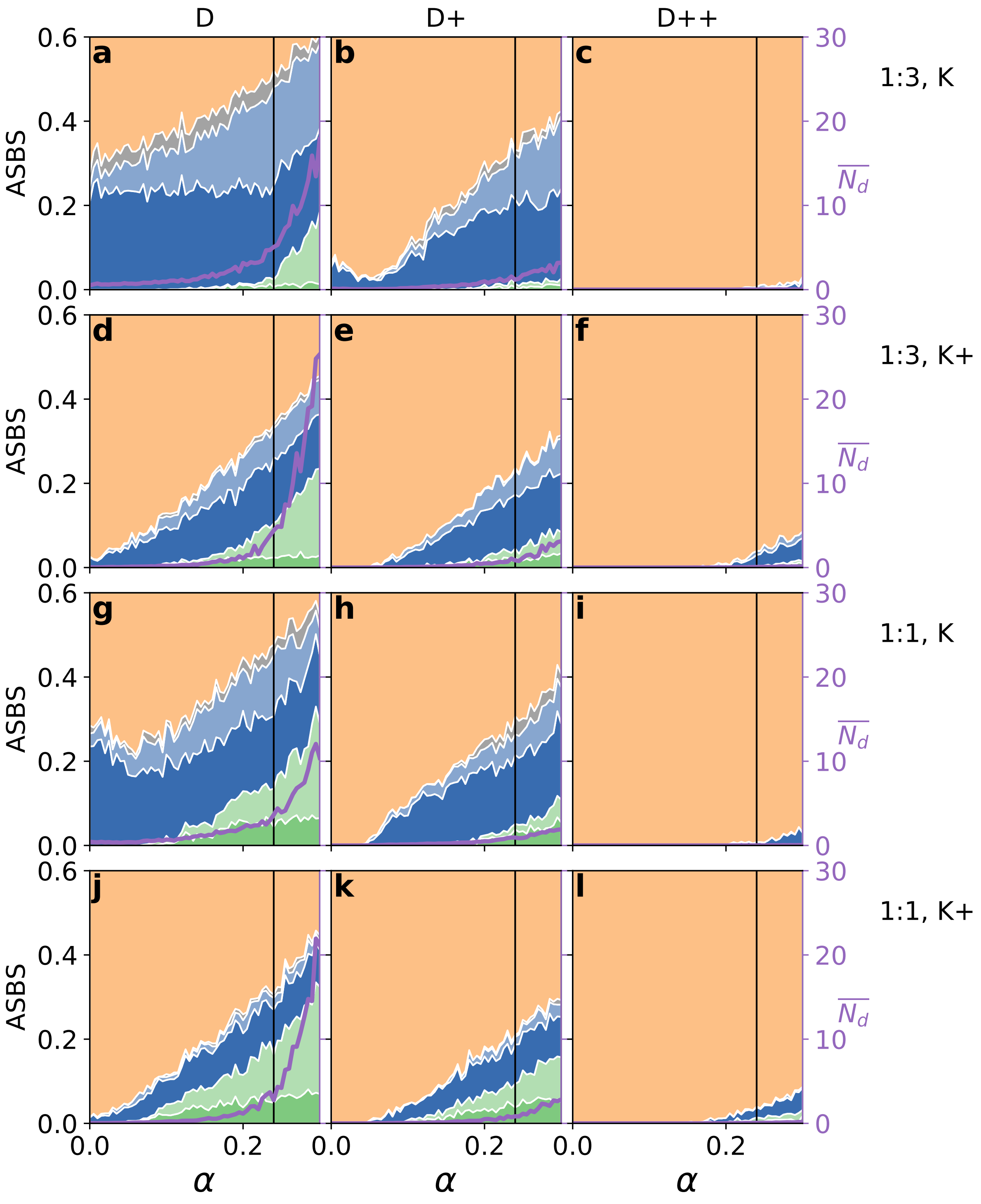

Supplementary Figure 3: **Average single node basin stability for parameter regimes of the Scandinavian grid.** The panels depict the ASBS for different asymptotic states as a function of α: phase synchronisation (■), normal 1-solitary (■) exotic 1-solitary (■), multiple normal solitaries (■), asymptotic states containing at least one exotic 1-solitary (■) and others not further distinguished (■). The columns refer to **a-j** standard (D) **b-k** strongly (D+) and **c-l** very strongly damped (D++) regimes. The first two rows refer to the status-quo scenario 1:3 with **a-c** standard (K) and **d-f** strongly coupled regime. The last two rows refer to the prosumer scenario 1:1 with **g-i** standard (K) and **j-l** strongly coupled regime. The right axis in each subfigure shows the expected number of desynchronised nodes $\overline{N_d}$. For each value of α, the shares sum up to 1. Note the linear scale of the ordinate and the cut-off at 0.6 to better visualise small values.

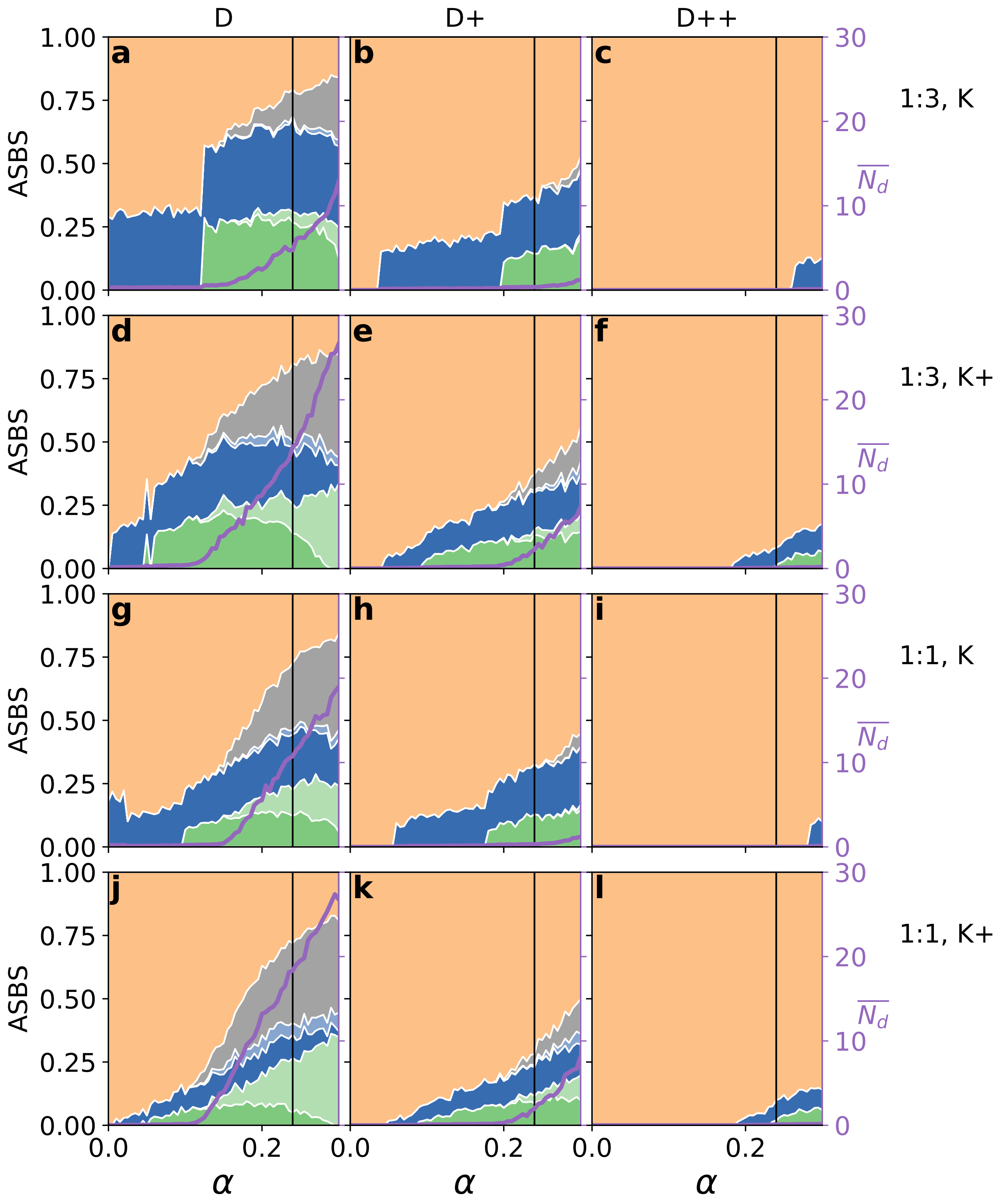

Supplementary Figure 4: **Average single node basin stability for parameter regimes of the circle topology.** The panels depict the ASBS for different asymptotic states as a function of α: phase synchronisation (■), normal 1-solitary (■) exotic 1-solitary (■), multiple normal solitaries (■), asymptotic states containing at least one exotic 1-solitary (■) and others not further distinguished (■). The columns refer to **a-j** standard (D) **b-k** strongly (D+) and **c-l** very strongly damped (D++) regimes. The first two rows refer to the status-quo scenario 1:3 with **a-c** standard (K) and **d-f** strongly coupled regime. The last two rows refer to the prosumer scenario 1:1 with **g-i** standard (K) and **j-l** strongly coupled regime. The right axis in each subfigure shows the expected number of desynchronised nodes $\overline{N_d}$. For each value of α, the shares sum up to 1. Note the linear scale of the ordinate.

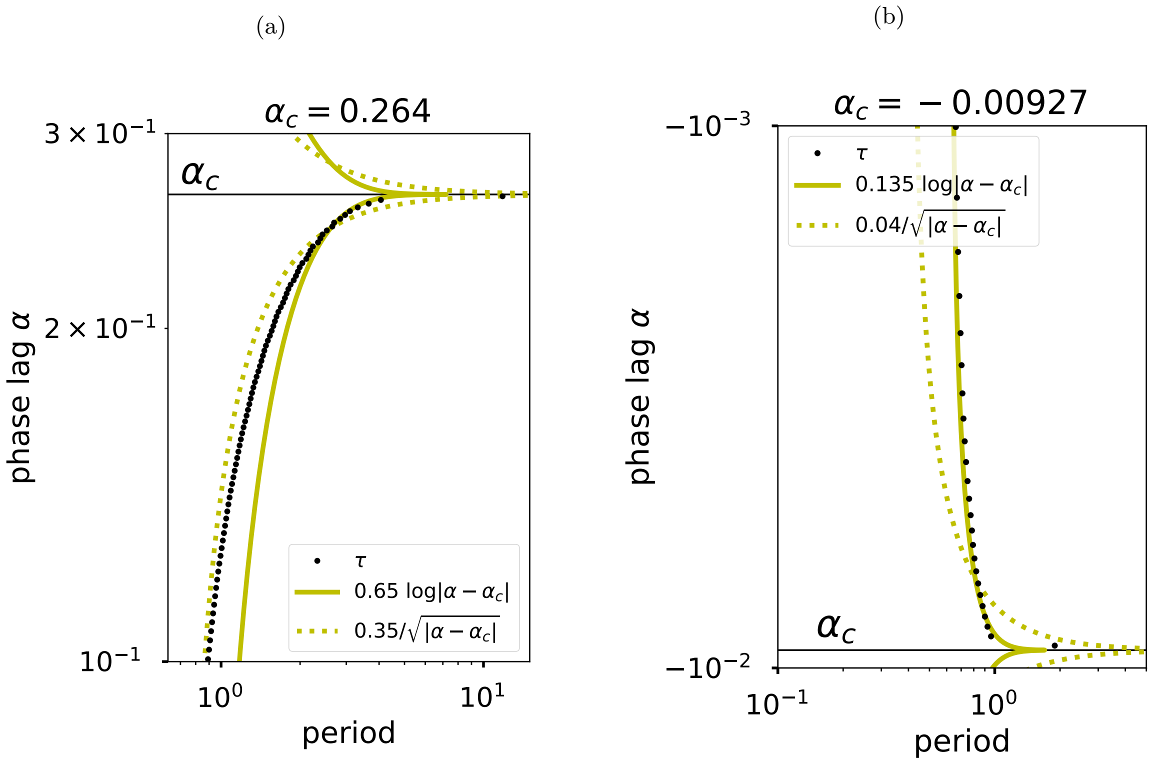

Supplementary Figure 5: **Bifurcation analysis.** **a** Bifurcation analysis of the infinite bus model with above parameters. **b** Bifurcation analysis of the circle model with standard parameters and control. **a**,**b** The yellow lines depict logarithmic and square root scaling laws with suitable parameters as visual guidance.

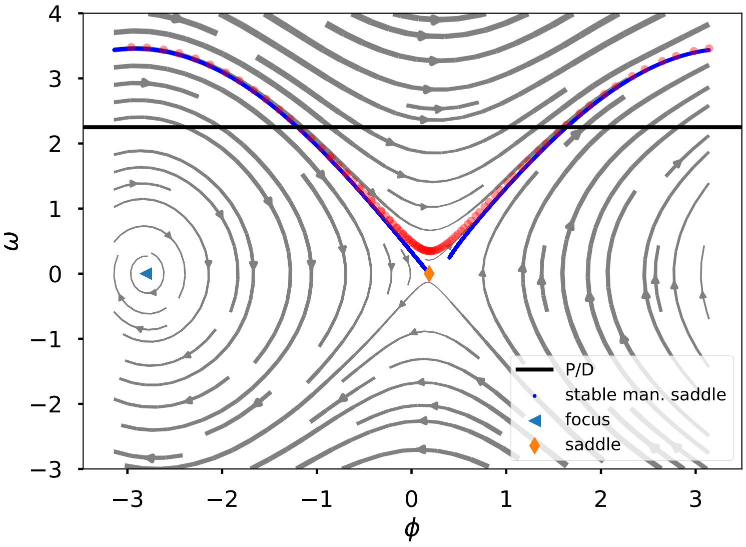

Supplementary Figure 6: **Bifurcation analysis.** Phase space of the infinite bus model $P = 1$, $D = 0.1$, $K = 2$, $H = 1$. The positions of the stable focus, the saddle and its stable manifold are indicated. The horizontal black line indicates the value of ω'_{lc}. See the text for further discussion.

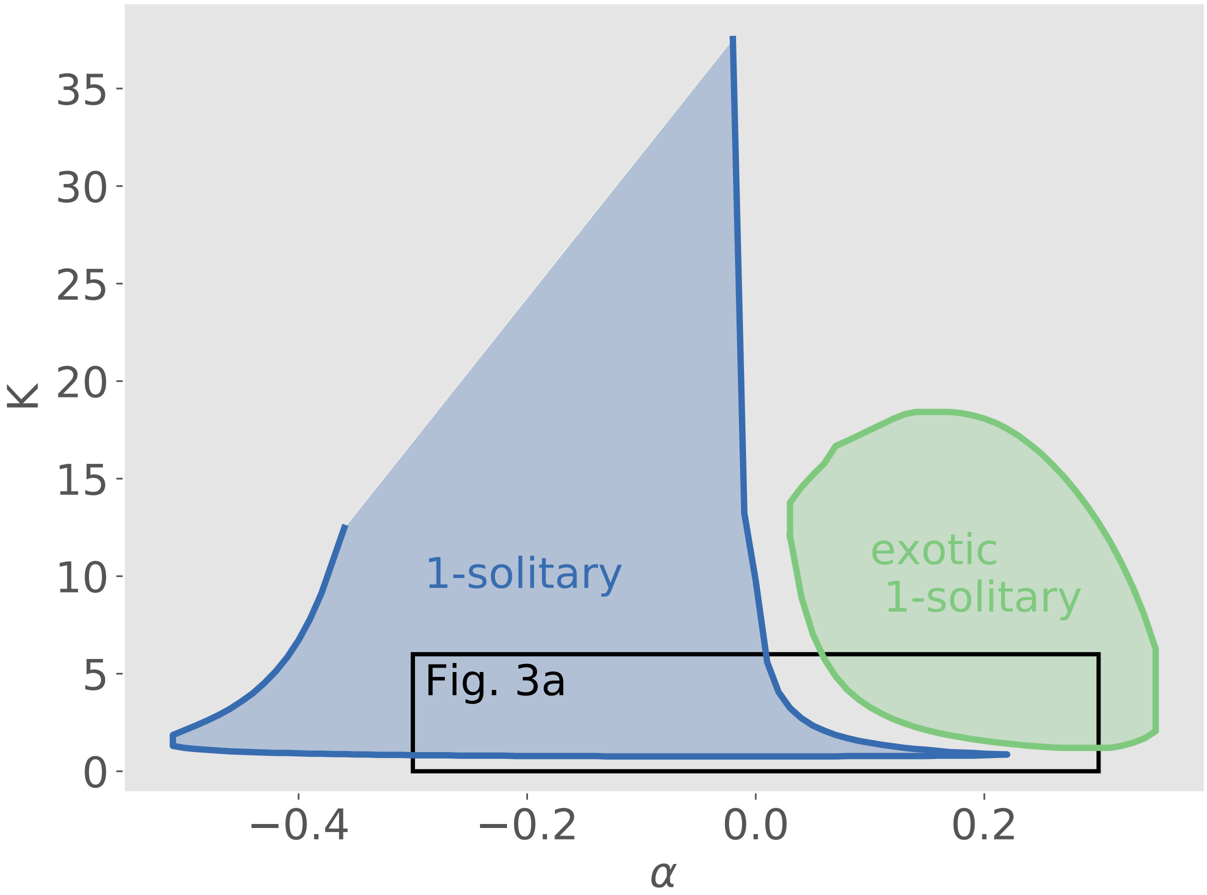

Supplementary Figure 7: **Continuation study of the 1-solitary state.** This figure shows the results of the continuation study of the 1-solitary state. While exotic solitaries eventually cease to exist (green boundary), 1-solitaries exist up to all studied coupling strengths (blue boundary). The black box indicates the region in Fig. 3.

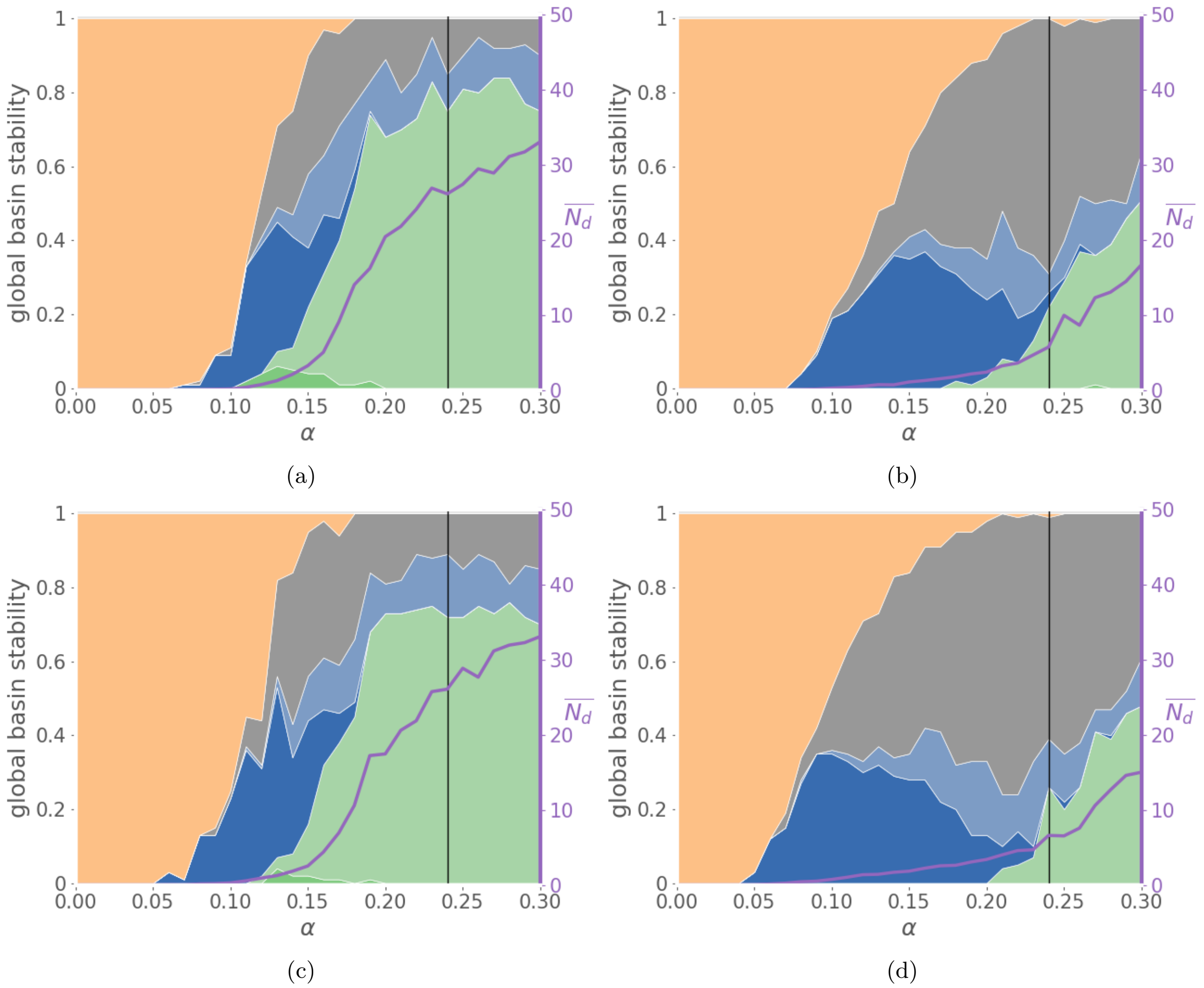

Supplementary Figure 8: **Asymptotic state resulting from whole network perturbations on the circle topology.** Variation of global basin stability of the asymptotic states (see Fig. 2) for different dynamical regimes of the circular topology vs. phase lag α. For each α, we draw 100 perturbations with $\Delta\omega = 1$. The upper two panels show the prosumer scenario (D+) with (**a**) standard (K) and (**b**) strong coupling (K+). The lower two panels show the status quo scenario (D+) with (**c**) standard (K) and (**d**) strong coupling (K+). The right axis in each subfigure shows the expected number of desynchronised nodes $\overline{N_d}$.

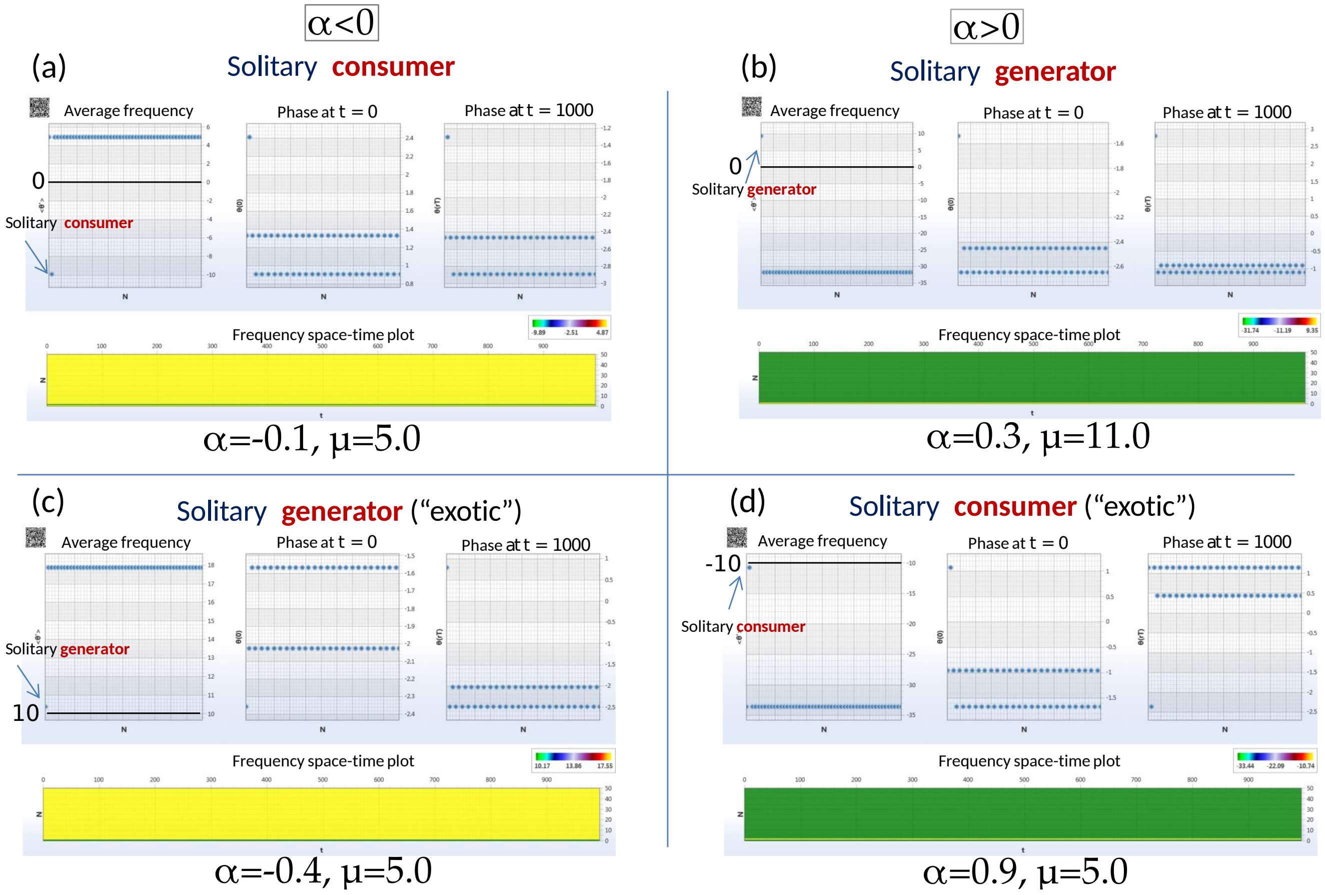

Supplementary Figure 9: **1-solitary states in mean-field coupled model** (Eqn. 12). Mean frequency and two phase snapshots are shown above. The frequency space-time plot is below. All simulation start from random initial conditions, the behaviour is shown after transient $T = 10^4$ time units. Parameters as in the main text. (Full resolution in electronic version.)

II. SUPPLEMENTARY NOTES

1. Homoclinic bifurcation of solitary oscillations and a continuation study

As it can be seen for instance in Fig. 3, there are regions in the $\alpha - K$ plane for which solitary oscillations do not exist. Our numerical studies give a strong indication that the underlying mechanism is a homoclinic bifurcation of the solitary limit cycle towards synchronisation. Furthermore, this is also suggested by our analogy with the single machine infinite bus model. The numerical procedure is as follows: the period of the solitary oscillation is estimated as the inverse time average of the phase velocity ω_s at the solitary node with index s:

$$\tau = \frac{2\pi}{\overline{\omega_s}} = 2\pi T \left(\int_0^T dt\, \omega_s(t) \right)^{-1} \tag{1}$$

for some large enough T such that we can be sure to measure several periods. Varying the phase lag α as the control parameter, we have used the period τ estimated at one parameter value to set $T = 10\tau$ for the next (updated) parameter value. The numerical integration is performed using SciPy's `CVode` solver with step size 10^{-3}.

In the case of a homoclinic bifurcation, it is expected that period τ scales logarithmically with the parameter in proximity to the bifurcation point α_c, i.e. $\tau \simeq -\log|\alpha_c - \alpha|$ (see for instance [1]).

Consider the phase portrait of the infinite bus model ($P = 1$, $D = 0.1$, $K = 2$, $H = 1$) close to the bifurcation point at $\alpha_c \approx 0.263$ pictured in Supplementary Figure 6. The red circles correspond to 100 initial conditions equally spaced on the limit cycle. Firstly, the locus of the limit cycle is close to the stable manifold of the saddle point (determined by backward integration) before they merge into a homoclinic orbit at α_c. Secondly, the distribution of points clusters near the saddle, indicating an increase of τ close to α_c due to a slowing down of trajectories. The numerically determined values of τ are given in Supplementary Figure 5a. As a guidance to the eye, the solid yellow line depicts a logarithmic scaling as expected for the homoclinic bifurcation in the infinite bus model. For comparison, the dashed yellow line gives a square root scaling in the case of an infinite-period bifurcation. It appears that close to α_c the logarithmic function is a better candidate for the scaling $\tau(\alpha)$, the onset of the divergence is much steeper than expected for the square root case. Analogously, $\tau(\alpha)$ is estimated for the circle model, here for the standard parametrisation and control. From Fig. 3 at $K = 6$, one expects a bifurcation at slightly negative $\alpha_c \approx -0.009$ from normal solitary oscillations to synchronisation. Repeating our analysis from the infinite bus model for the circle indeed strongly suggests a homoclinic bifurcation (Supplementary Figure 5b). In summary, our numerical investigations and the analogy to the infinite bus approximation support the hypothesis that the global bifurcation curves in the parameter plane of the circle model are homoclinic.

Finally we give the results of a continuation study of the solitary states in Supplementary Figure 7. Note in particular that the region in which solitaries exist seems to continue to arbitrarily high coupling strength.

2. Global basin stability

Formally, the basin stability [2–4] and [5] $S_\mathcal{B}$ of attractor $\mathcal{A}$ with basin of attraction $\mathcal{B}(\mathcal{A})$ is defined as

$$\begin{aligned} S_\mathcal{B} &:= \mu\left(\mathcal{B}(\mathcal{A})\right) \\ &= \int_{R \subseteq X} \mathbb{I}_{\mathcal{B}(\mathcal{A})}\, d\mu = \int_{R \subseteq X} \mathbb{I}_{\mathcal{B}(\mathcal{A})}(x)\rho(x)\, dx^d\ , \end{aligned} \tag{2}$$

where $\mathbb{I}_{\mathcal{B}(\mathcal{A})}(x)$ is the indicator function of the basin giving 1 if $x \in \mathcal{B}(\mathcal{A})$ and 0 otherwise.

In this definition, $S_\mathcal{B}$ depends on the choice of a compact subset $R \subseteq X$ of the d-dimensional phase space X (except X is bounded, e.g. a Torus) and the selection of a probability measure μ on R, typically in terms of probability density $\rho(x)$. ρ can be suitably chosen to incorporate the knowledge about the statistical nature of perturbations, for instance short circuit durations in power grids.

Set R can then be parametrised by a maximum frequency deviation $\Delta\omega$ around the synchronous state ϕ^*:

$$\begin{aligned} R(\Delta\omega) = \{(\phi, \omega) \in X\ &: \forall_k\ \phi_k^* - \pi \leq \phi_k \leq \phi_k^* + \pi \\ &\wedge \forall_k\ |\omega_k| \leq \Delta\omega\}\ . \end{aligned} \tag{3}$$

We refer to $S_{\mathcal{B}}$ as global basin stability because it can be interpreted as applying a perturbation to every node. If the perturbation at each node has an independent fixed probability to destabilize the system, this means that $S_{\mathcal{B}}$ becomes exponentially small as one increases the number of nodes. Therefore, care needs to be taken when interpreting the results. In the Scandinavian topology it means that once a small fraction of nodes becomes vulnerable to perturbations, the global basin stability will be almost zero and lost in the sampling error. On the other hand the average single node basin stability that we consider in the paper uses perturbations that are local on the network. The likelihood to destabilize the system is thus proportional to the number of vulnerable nodes. Tracking how additional localized asymptotic states come in and cause more vulnerability in the network is thus more robustly done by using ASBS.

For the circle topology, where the system is homogeneous, all nodes become vulnerable at the same time. We show the dramatic reduction in the global basin stability ($\Delta\omega = 1$) that results in Figure 8. Notably, even here solitaries play a role.

As mentioned above and discussed in the Methods section, the main tool of our investigation is based on network-local perturbations and termed average single-node basin stability, ASBS. For completeness, we node that this version of basin stability can be written as above by constructing the spaces R_k which consist of the states which satisfy:

$$\begin{aligned} \phi_i &= \phi_i^* && \forall i \neq k \\ \omega_i &= \omega_{\text{global}} && \forall i \neq k \end{aligned}$$

where ϕ^* and ω_{global} give the synchronous state. The perturbation space is then given by

$$R = \bigcup R_k \,. \tag{4}$$

And the measure we chose is homogeneous within the region $|\omega| < \Delta\omega$.

III. SUPPLEMENTARY DISCUSSION

Analytic analysis of the circle model of the power grid

In this further discussion we consider an abstract power grid organisation, the so-called *circle model*, which demonstrates the main features we find responsible for prompt desynchronisation. In this model, the desired synchronised state exists and is stable. However, the model itself appears to be genuinely multistable. There arise indeed many other, the so-called *solitary states* in which one or a few power grid units start to behave differently with respect to all others perfectly synchronised such that not only the phase but also the average frequency deviates from the synchronous behaviour. Remarkably, these solitary states are Lyapunov stable too and hence, they cannot be excluded from the global system dynamics even by an essential increase of the inner network connectivity (as it would be naturally expected). Furthermore, it is found that this kind of network multistability arises under realistic operating parameters applied to power grids. If so, there is always a danger that sudden non-small disturbances associated e.g. with fluctuations in production or demand may trigger desynchronisation of one or several power generators/consumers even if the synchronous state is perfectly operating in a stable regime. While our model is topologically simplified and does not account for complication of the real power grid structure, we show through extensive numerical experiments that our predictions remain robust when including these effects for the Scandinavian power grid.

We define the circle power grid as follows (see also Supplementary Figure 1b). Let it contains N units and let a half of them, $N/2$, be identical generators (representing power plants) and the other half identical motors (representing consumers). Place all of them on a ring with the "alternative" disposition and assume that each unit is connected with R of its nearest neighbours to the left and to the right, and with an equal coupling strength K. Supplementary Figure 1b shows the case of $R = 2$ which we have used for our numerical explorations. In the case that all units obey the same type of equation of motion, the Kuramoto model with inertia, of the form

$$\begin{aligned} \ddot{\phi}_i + \epsilon\dot{\phi}_i = \\ (-1)^{i+1}\Omega - 2RK\sin\alpha + K \sum_{j=i-R, j\neq i}^{i+R} \sin(\phi_j - \phi_i + \alpha) \end{aligned} \tag{5}$$

($i = 1, \ldots, N$), where the term $K\sin\alpha$ shifts the oscillator eigenfrequency Ω up or down depending on K and the parameter α.

Here, we use the notation

$$\begin{aligned}\Omega &:= |P_i|/H,\ \ \forall\, i\\ \epsilon &:= D_i/H,\ \ \forall\, i\\ K &:= K_{ij}/H,\ \ \forall\, i,j\ .\end{aligned}$$

In the lossless case $\alpha = 0$ this term disappears and one comes to the classical Kuramoto model with bi-modal frequency distribution

$$\ddot{\phi}_i + \epsilon\dot{\phi}_i = (-1)^{i+1}\Omega + K\sum_{j=i-R}^{i+R}\sin(\phi_j - \phi_i) \tag{6}$$

which dynamics is comparably simpler. Introducing non-zero α, however, makes the global network behaviour more sensitive to desynchronisation due to multiple appearance of solitary states, not avoidable even with large increase of the coupling strength K.

a. Synchronous state of the circle model To identify a synchronous state of the circle power grid model (Eqn. 5), re-write it in a two-group form denoting the generators by θ_i and the consumers $\psi_i,\ i = 1,\ldots,N/2$:

$$\begin{aligned}\ddot{\theta}_i + \epsilon\dot{\theta}_i &= +\Omega - 2RK\sin\alpha + K\sum_{j=i-R, j\neq i}^{i+R}\sin(\theta_j - \theta_i + \alpha),\\ \ddot{\psi}_i + \epsilon\dot{\psi}_i &= -\Omega - 2RK\sin\alpha + K\sum_{j=i-R, j\neq i}^{i+R}\sin(\psi_j - \psi_i + \alpha).\end{aligned} \tag{7}$$

Assume $\theta_1 = \cdots = \theta_{N/2} \equiv \theta$ and $\psi_1 = \cdots = \psi_{N/2} \equiv \psi$, and subtract the second equation from the first one. The following equation is obtained for the phase difference $\eta = \theta - \psi$:

$$\ddot{\eta} + \epsilon\dot{\eta} = 2\Omega - RK(\sin(\eta + \alpha) + \sin(\eta - \alpha)), \tag{8}$$

By simple trigonometry, it can be re-written as

$$\ddot{\eta} + \epsilon\dot{\eta} = 2\Omega - \mu\cos\alpha\sin\eta, \tag{9}$$

where a new coupling parameter $\mu = 2RK$ is introduced. This is an equation of motion in the two-dimensional invariant manifold indicated by the conditions $\theta_1 = \cdots = \theta_{N/2} \equiv \theta$, $\psi_1 = \cdots = \psi_{N/2} \equiv \psi$ as above. Synchronous states of the original N-dim Eqn. 5 are given by the roots of its right-hand side, which are

$$\eta_O = \arcsin\frac{2\Omega}{\mu\cos\alpha}, \qquad \eta_S = \pi - \arcsin\frac{2\Omega}{\mu\cos\alpha} \tag{10}$$

corresponding to stable node $O = (\eta_O; 0)$ and saddle $S = (\eta_S; 0)$ of the in-manifold scalar Eqn. 9. The states are born in a saddle-node bifurcation at the bifurcation curve

$$\mu_{sync} = \frac{2\Omega}{\cos\alpha}, \tag{11}$$

and they exist for all $\mu > \mu_{sync}$, where node O transforms into a stable focus soon after the bifurcation at $\mu_{foc} = \frac{2\Omega}{\cos\alpha}\sqrt{1 + \epsilon^4/64\Omega^2}$.

The synchronous state we are looking for is given by the stable equilibrium O. The in-manifold stability, however, does not guarantee its transverse stability. Our numerics indicate, however, that the equilibrium O is also stable in the whole N-dimensional space of the original model Eqn. 5.

We conclude that the synchronous state of the circle model (Eqn. 5) is born in a saddle-node bifurcation as the coupling strength μ exceeds the bifurcation value μ_{sync} and it preserves the stability with further increase of μ. In

the state, generators and consumers create two phase-synchronised groups with the phase shift between them equal η_O. At the saddle-node bifurcation curve $\mu = \mu_{sync}$, the phase shift is $\eta_O = \pi/2$ and it decreases to zero as $\mu \to \infty$. Frequency Ω_O of the synchronous state O is obtained from Eqn. 5 as:

$$\begin{aligned}\Omega_O &= \frac{\mu \sin\alpha}{2\epsilon}\left(\sqrt{1-\left(\frac{2\Omega}{\mu\cos\alpha}\right)^2}-1\right)\\ &= \frac{\mu\sin\alpha}{2\epsilon}\left(\cos\eta_O - 1\right).\end{aligned}$$

It follows therefore that synchronous frequency Ω_O is zero in the lossless case $\alpha = 0$, negative for $\alpha > 0$ and positive for $\alpha < 0$.

b. Solitary states in mean-field coupled circle model Solitary states can be derived analytically in the case of mean-field approximation of the circle model, i.e. when $R = N/2$ in Eqn. 5. For convenience, we write the mean-field model in the standard normalised form

$$\ddot{\phi}_i + \epsilon\dot{\phi}_i = (-1)^{i+1}\Omega + \frac{\mu}{N}\sum_{j=1}^{N}\sin(\phi_j - \phi_i - \alpha)\ . \tag{12}$$

Here parameter α can be positive or negative. Due to the assigned symmetry, these two cases are reducing explicitly to each other by simple transformation $\alpha \to -\alpha$, $\Omega \to -\Omega$, $\phi_i \to -\phi_i$, at which the generators and consumers exchange their network disposition. Therefore, the system dynamics appears to be equivalent for $\alpha < 0$ and $\alpha > 0$ and if so, only one of the cases should be derived. The symmetry property is also valid for the general, non mean-field form of the circle power grid model (Eqn. 5), which is clearly demonstrated by our simulations. However, it is not the case for non-symmetric generator/consumer disposition as it is normally the case in real power grid networks, e.g. in the Scandinavian power grid.

The synchronous state O of the mean field-model Eqn. 12 is obtained by the same procedure as in the previous section applied to the two-group representation Eqn. 7 including generators θ_i and consumers ψ_i:

$$\begin{aligned}&\ddot{\theta}_i + \epsilon\dot{\theta}_i = \\ &\qquad + \Omega + \frac{\mu}{N}\sum_{j=1}^{N/2}(\sin(\theta_j - \theta_i - \alpha) + \sin(\psi_j - \theta_i - \alpha)),\\ &\ddot{\psi}_i + \epsilon\dot{\psi}_i = \\ &\qquad - \Omega + \frac{\mu}{N}\sum_{j=1}^{N/2}(\sin(\psi_j - \psi_i - \alpha) + \sin(\theta_j - \psi_i - \alpha)).\end{aligned}$$

So, it has the form

$$O:\ \theta_1 = \cdots = \theta_{N/2} \equiv \theta,\quad \psi_1 = \cdots = \psi_{N/2} \equiv \psi \tag{13}$$

with a constant phase shift $\eta_O = \theta - \psi$ between generators and consumers, where $\eta_O = \arcsin\frac{2\Omega}{\mu\cos\alpha}$. The synchronous state exists and is stable for all μ above the bifurcation curve as in 11.

Beyond the existence of synchronous state O which is phase and frequency synchronised, numerous solitary states of different configurations are typically arising in the mean-field model (Eqn. 12). They consist normally of several phase clusters, each rotating with its own frequency, manifesting in such a way a phenomenon of *frequency clustering.* This peculiar phenomenon differs from usual phase clustering well-known in (pure) phase oscillatory networks, i.e. the standard Kuramoto model. De facto, due to the presence of inertia, the system dynamics becomes much more involved. The main peculiarity is an enormous multistability, i.e., when the number of co-existing stable solitary and other states can grow exponentially with the system size N, moreover, in an essential domain of the system parameters. If so, *spatial chaos* [6–8] becomes an inherent characteristic of the model. We are coming apparently to a new reality, still not comprehended, in the field of coupled oscillators with inertia.

The simplest but not trivial solitary-type behaviour is caused by the appearance of a 1-solitary state. Here just one generator or consumer – let for definiteness it be a generator θ_1 .- splits off from the others, which are frequency synchronised consisting of two phase clusters: one of remaining generators and the other of all consumers, i.e.,

$$\Phi_{1-sol}: \ \theta_1 \equiv \gamma; \ \theta_2 = \cdots = \theta_{N/2} \equiv \theta, \ \psi_1 = \cdots = \psi_{N/2} \equiv \psi. \tag{14}$$

The examples of four different 1-solitary states for the mean-field model (Eqn. 12) are illustrated in Supplementary Figure 9, for solitary generator (b,c) and consumer (a,d). Due to the model symmetry, the case with solitary consumers can be reduced to the generator one by simple parameter/variable change $\alpha \to -\alpha$, $\Omega \to -\Omega$, $\phi_i \to -\phi_i$. We skip it in the section and concentrate further on the states with the solitary generator as in Supplementary Figure 9.

As it is illustrated in Supplementary Figure 9, there are two types of 1-solitary states in which a generator is detached. Actually, what happens in both cases is that the generator continues to rotate close to its eigenfrequency $\Omega = 10$ in the situation when all others units synchronise with smaller (b) or larger (c) mean frequency. The first situation (b) may be considered as a ”normal”, as the desirable synchronous frequency should naturally be less then these of the generators and it equals zero if $\alpha = 0$ and deviates to negative values as $\alpha > 0$. Another 1-solitary state shown in (c) obeys a counter-intuitive property: the mean frequency of the synchronous cluster appears to be larger than 10. We call this state an ”exotic” and it exists for negative α only.

The parameter region for both, ”normal” and ”exotic” 1-solitary states (with a generator detached) are shown in Supplementary Figure 7. The analogous regions for the case with a consumer detached (see examples in Supplementary Figure 9 (a,d)) are symmetrically disposed with respect to the $\alpha = 0$ axis.

c. Reduction to solitary manifold In the new variables (γ, θ, ψ) for 1-solitary state (Eqn. 14) the mean-field model (Eqn. 12) consists of only three equations:

$$\begin{aligned}
&\ddot{\gamma} + \epsilon\dot{\gamma} = \\
&\qquad + \Omega + \frac{\mu}{N}\left(\left(\frac{N}{2} - 1\right)\sin(\theta - \gamma - \alpha)\right. \\
&\qquad \left. + \ \frac{N}{2}\sin(\psi - \gamma - \alpha) + \sin(-\alpha)\right), \\
&\ddot{\theta} + \epsilon\dot{\theta} = \\
&\qquad + \Omega + \frac{\mu}{N}\left(\frac{N}{2}\sin(\psi - \theta - \alpha) + \sin(\gamma - \theta - \alpha)\right. \\
&\qquad \left. + \left(\frac{N}{2} - 1\right)\sin(-\alpha)\right), \\
&\ddot{\psi} + \epsilon\dot{\psi} = \\
&\qquad - \Omega + \frac{\mu}{N}\left(\left(\frac{N}{2} - 1\right)\sin(\theta - \psi - \alpha)\right. \\
&\qquad \left. + \sin(\gamma - \psi - \alpha) + \frac{N}{2}\sin(-\alpha)\right).
\end{aligned}$$

This is a reduced model of three coupled virtual ”pendula” governing the dynamics in the respective 6-Dim solitary manifold. The pendula are linear but the coupling terms, however, are strongly non-linear (contain sinusoidal functions). This results in complex dynamical behaviour characterised by multiple solitary states and chaos. As coupling terms depend only on the phase differences, the system dimension can be further reduced by introducing phase difference variables $\nu = \gamma - \psi$, $\eta = \theta - \psi$. In the new variables, only two equations are left:

$$
\begin{aligned}
&\ddot{\nu} + \epsilon\dot{\nu} = \\
&\qquad 2\Omega + \frac{\mu}{2}\left(\left(1 - \frac{2}{N}\right)\sin\alpha - \sin(\nu+\alpha) - \frac{2}{N}\sin(\nu-\alpha)\right. \\
&\qquad \left. + \left(1-\frac{2}{N}\right)\left(\sin(\eta-\nu-\alpha) - \sin(\eta-\alpha)\right)\right), \\
&\ddot{\eta} + \epsilon\dot{\eta} = \\
&\qquad 2\Omega + \frac{\mu}{N}\sin\alpha - \frac{\mu}{2}\left(\sin(\eta+\alpha) + \left(1-\frac{2}{N}\right)\sin(\eta-\alpha)\right) \\
&\qquad + \frac{\mu}{N}\left(\sin(\nu-\eta-\alpha) - \sin(\nu-\alpha)\right).
\end{aligned}
\tag{15}
$$

This is a system of only two coupled "pendula", maintaining on a 4-Dim invariant manifold of the whole N-dimensional phase space. It is complicated for analytic study, as each pendulum as well as all coupling terms are non-linear. However, the model is low-dimensional so it can be easily derived in a standard way with the use of numerical simulations to identify 1-solitary states for the N-dimensional mean-field model (Eqn. 12), up to the thermodynamic limit $N \to \infty$. Note, that all in-manifold regimes which develop within the framework of the reduced system (Eqn. 15) should be tested on the transverse stability with respect to out-of-manifold perturbations, to guarantee the existence in the whole N-dimensional model.

This approach is also applicable to k-solitary states, with any $k = 2, 3..$, of the form

$$
\begin{aligned}
\Phi_{k-sol} : &\ \theta_1 = \cdots = \theta_k \equiv \gamma \\
&\ \theta_{k+1} = \cdots = \theta_{N/2} \equiv \theta,\ \ \psi_1 = \cdots = \psi_{N/2} \equiv \psi.
\end{aligned}
\tag{16}
$$

Reduced low-dimensional systems for the k-solitary states can be obtained in a similar way as in the $k = 1$ case above. They are slightly different with the system parameters however, their dimensions are the same. Note finally, that the behaviour of solitary states becomes even more puzzled if the synchronised elements, variables θ_i and ψ_i in (Eqn. 16), split off by more than two clusters. The above reduced procedure still works, however, the dimension of the "solitary" manifold becomes larger then 4. Nevertheless, the study in this case is yet much simpler than the original N-dimensional model.

[1] Strogatz, S. H. *Nonlinear Dynamics and Chaos: With Applications to Physics, Biology, Chemistry, and Engineering (Studies in Nonlinearity)* (CRC Press, 2018).

[2] Menck, P. J., Heitzig, J., Marwan, N. & Kurths, J. How basin stability complements the linear-stability paradigm. *Nat. Phys.* **9**, 89 (2013).

[3] Menck, P. J., Heitzig, J., Kurths, J. & Schellnhuber, H. J. How dead ends undermine power grid stability. *Nat. Comm.* **5**, 3969 (2014).

[4] Schultz, P., Menck, P. J., Heitzig, J. & Kurths, J. Potentials and limits to basin stability estimation. *New Journal of Physics* **19**, 023005 (2017).

[5] Schultz, P. *Stability Concepts of Networked Infrastructure Networks.* Ph.D. thesis, Humboldt-Universität zu Berlin, Mathematisch-Naturwissenschaftliche Fakultät (2018).

[6] Elphick, C., Coullet, P. & Repaux, D. Nature of spatial chaos. *Phys. Rev. Lett* **58**, 431–434 (1987).

[7] Nizhnik, L. P., Nizhnik, I. L. & Hasler, M. Stable Stationary Solutions in Reaction–Diffusion Systems Consisting of a 1-D Array of Bistable Cells. *International Journal of Bifurcation and Chaos* **12**, 261–279 (2002).

[8] Omelchenko, I., Maistrenko, Y., Hövel, P. & Schöll, E. Loss of coherence in dynamical networks: Spatial chaos and chimera states. *Phys. Rev. Lett.* **106** (2011).
